# HIF2α activation and mitochondrial deficit due to iron chelation cause retinal atrophy

**DOI:** 10.15252/emmm.202216525

**Published:** 2023-01-16

**Authors:** Yang Kong, Pei‐Kang Liu, Yao Li, Nicholas D Nolan, Peter M J Quinn, Chun‐Wei Hsu, Laura A Jenny, Jin Zhao, Xuan Cui, Ya‐Ju Chang, Katherine J Wert, Janet R Sparrow, Nan‐Kai Wang, Stephen H Tsang

**Affiliations:** ^1^ Department of Ophthalmology, Vagelos College of Physicians and Surgeons Columbia University New York NY USA; ^2^ Department of Ophthalmology Kaohsiung Medical University Hospital, Kaohsiung Medical University Kaohsiung Taiwan; ^3^ School of Medicine, College of Medicine Kaohsiung Medical University Kaohsiung Taiwan; ^4^ Institute of Biomedical Sciences National Sun Yat‐sen University Kaohsiung Taiwan; ^5^ Department of Biomedical Engineering, The Fu Foundation School of Engineering and Applied Science Columbia University New York NY USA; ^6^ Departments of Ophthalmology and Molecular Biology University of Texas Southwestern Medical Center Dallas TX USA; ^7^ The Hamon Center for Regenerative Science and Medicine University of Texas Southwestern Medical Center Dallas TX USA; ^8^ Jonas Children's Vision Care, and Bernard and Shirlee Brown Glaucoma Laboratory, Columbia Stem Cell Initiative, Pathology and Cell Biology, Institute of Human Nutrition, Vagelos College of Physicians and Surgeons Columbia University New York NY USA

**Keywords:** HIF2α upregulation, iron deficiency, mitochondrial deficit, RPE atrophy, α‐ketoglutarate, Metabolism, Signal Transduction

## Abstract

Iron accumulation causes cell death and disrupts tissue functions, which necessitates chelation therapy to reduce iron overload. However, clinical utilization of deferoxamine (DFO), an iron chelator, has been documented to give rise to systemic adverse effects, including ocular toxicity. This study provided the pathogenic and molecular basis for DFO‐related retinopathy and identified retinal pigment epithelium (RPE) as the target tissue in DFO‐related retinopathy. Our modeling demonstrated the susceptibility of RPE to DFO compared with the neuroretina. Intriguingly, we established upregulation of hypoxia inducible factor (HIF) 2α and mitochondrial deficit as the most prominent pathogenesis underlying the RPE atrophy. Moreover, suppressing hyperactivity of HIF2α and preserving mitochondrial dysfunction by α‐ketoglutarate (AKG) protects the RPE against lesions both *in vitro* and *in vivo*. This supported our observation that AKG supplementation alleviates visual impairment in a patient undergoing DFO‐chelation therapy. Overall, our study established a significant role of iron deficiency in initiating DFO‐related RPE atrophy. Inhibiting HIF2α and rescuing mitochondrial function by AKG protect RPE cells and can potentially ameliorate patients' visual function.

## Graphical abstract

Deferoxamine (DFO) stabilizes HIF2α and disrupts mitochondrial oxidative phosphorylation, leading to RPE atrophy. Inhibiting HIF2α and preserving mitochondrial oxidative phosphorylation by α‐ketoglutarate (AKG) mitigate RPE cell death, and ameliorate visual impairment in the clinic.

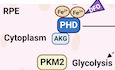

## Introduction

Iron is essential in various physiological processes across species, including oxygen transport, energy production and enzymatic catalysis. However, mounting evidence has revealed the consequences of iron excess in disrupting cellular homeostasis and causing cell death (Gozzelino & Arosio, 2016; Eid *et al*, 2017). Of note, free iron contributes to the production of reactive oxygen species (ROS) that trigger programmed cell death, as exemplified by ferroptosis, a newly‐identified form of cell death (Dixon *et al*, 2012). In the clinic, iron overload, as seen in transfusion‐dependent thalassemia patients, results in malfunction across tissues and organs (Kohgo *et al*, 2008; Shah *et al*, 2019). Chelation therapy is therefore, indispensable to reducing excess iron and minimizing its systemic toxicity. The advent of deferoxamine (DFO), an iron chelator, significantly improved the life expectancy of transfusion‐dependent thalassemia patients as it minimizes systemic complications linked to iron overload (Cohen *et al*, 1984; Brittenham *et al*, 1994; Borgna‐Pignatti *et al*, 2004; Poggiali *et al*, 2012). However, adverse effects of iron chelation emerged in patients with a history of taking DFO (Brittenham, 2011), which prompted a critical need to understand the delicate iron balance and its governing mechanisms in maintaining cell vitality for the clinical management of DFO toxicity.

Iron loss by chelation therapy mainly affects iron‐dependent signaling cascades, including mitochondrial oxygen consumption and hypoxia. Coupling oxygen consumption with electron transport is a major function of mitochondria for energy production, in which iron plays an indispensable role. Iron deficiency was reported to be linked to ROS production and profound disruption in mitochondrial respiration (Walter *et al*, 2002; Fujimaki *et al*, 2019). Moreover, a defective respiratory chain creates a hypoxic condition and induces activation of hypoxia inducible factor (HIF) α. HIFs are heterodimeric transcription factors that are mainly responsible for oxygen‐dependent reactions inside the cell (Wang *et al*, 1995). The stability and transactivation of HIFα rely on prolyl hydroxylase domain (PHD) protein‐mediated hydroxylation, in which Fe^2+^ and α‐ketoglutarate (AKG) function as key co‐factors. Supplementing Fe^2+^ or AKG was reported to suppress HIFα *in vitro* (Kaelin, 2005; Kaelin & Ratcliffe, 2008). As a master regulator of oxygen homeostasis and aerobic glycolysis, the HIFα system is implicated in multiple biological processes, including angiogenesis, extracellular matrix dynamics and cell survival. Abnormal HIFα due to genetic defects or extracellular cues contributes to congenital defects, inflammation, cardiovascular malfunctions and oncogenesis (Bertout *et al*, 2008; Majmundar *et al*, 2010). The HIFα family consists of three major isoforms in mammals: HIF1α and its paralog HIF2α, which overlap in structure, and HIF3α (Semenza, 2012). Unlike the ubiquitous expression of HIF1α, HIF2α is exclusively expressed by specific tissues, such as vascular endothelium, liver parenchyma, kidney epithelium, cornea, thymus, and cerebellar Purkinje cells (Talks *et al*, 2000). Despite the structural resemblance and functional redundancy between HIF1α and HIF2α, nuanced distinctions in transcriptional regulation have been noted (Ginouves *et al*, 2008; Mastrogiannaki *et al*, 2009; Keith *et al*, 2011; Downes *et al*, 2018). Understanding the differential impact of HIF1α and HIF2α in a cell‐specific manner in both healthy and diseased conditions is necessary to study their contribution to disease initiation and progression and explore their therapeutic potential.

Ophthalmic toxicity from DFO was first reported among patients who presented with neurosensory impairment (Davies *et al*, 1983; Olivieri *et al*, 1986; Rahi *et al*, 1986; Baath *et al*, 2008). Of interest, iron deficiency in the eye significantly undermines metabolic homeostasis of retinal pigment epithelial (RPE) cells (Kanow *et al*, 2017). Despite histological anomalies in the RPE, detailed pathological features of DFO‐related retinopathy and its molecular basis are yet to be defined. We, therefore, sought to investigate the influence of iron deficiency in retinal cells and explore a therapeutic solution to DFO‐related retinopathy. By inducing DFO toxicity in mice and cultured RPE derived from human induced pluripotent stem cells (iPSCs), we investigated the mechanism underpinning DFO‐related retinopathy. Our clinical and experimental characterization provides new evidence that the RPE is a primary target for DFO toxicity, which raises ROS levels and disturbs mitochondrial respiration. Additionally, we noted stabilization of HIF2α rather than HIF1α in response to DFO toxicity in RPE, which transcriptionally upregulates different clusters of genes pertaining to cell viability, glycolysis, and iron transport. Strikingly, AKG suppresses the hyperactivity of HIF2α and modifies metabolic anomalies associated with DFO toxicity in the RPE. Taken together, this study demonstrates the pathological consequences of iron depletion in RPE caused by DFO. It establishes a profound impact of mitochondrial dysfunction and upregulated HIF2α on RPE atrophy. Destabilizing HIF2α and preserving mitochondrial capacity by AKG prevents RPE cell death and can alleviate patient's visual decline due to DFO intake.

## Results

### Ophthalmic phenotyping of patients undergoing DFO treatment

**Figure 1:**
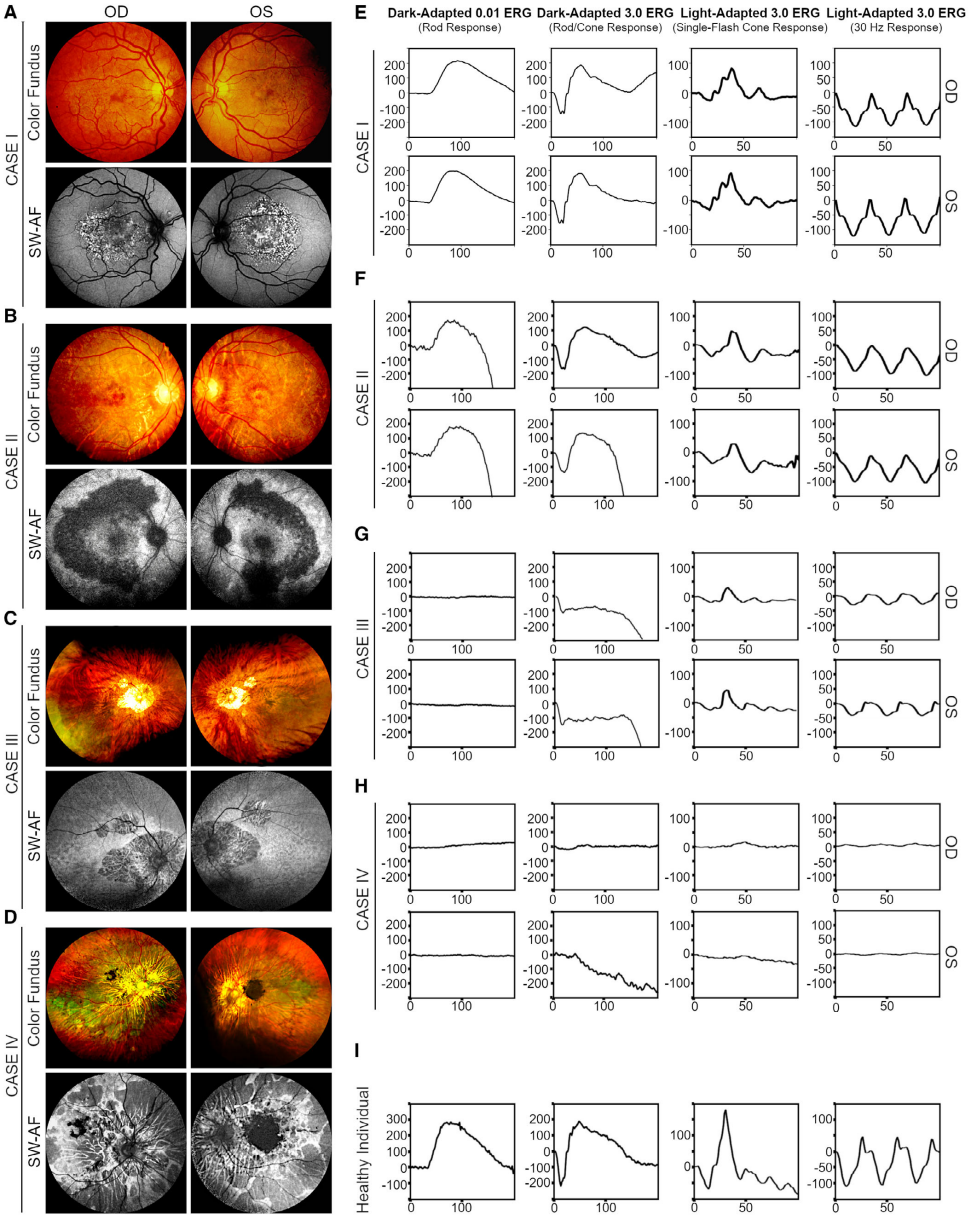
Ophthalmic examinations of chelation‐dependent thalassemia patients show degenerative changes and functional decline A–DColor fundus photographs and SW‐AF examinations on four patients of β‐thalassemia subject to chelation therapy by DFO for at least 16 years. Fundus phenotypes, including RPE mottling and depigmentation in the macula (Case I; Gelman *et al*, 2014), choroidal sclerosis in the perimacular areas (Case II), peripapillary (Cases III and IV), and subretinal pigmentation (Case IV) were evaluated. RPE lesions including concentric distribution of stippled hyper‐autofluorescence at the macula (Case I), large areas of hypo‐autofluorescent regions at parapapillary or perimacular area (Cases II and III), and extensive RPE loss in both eyes (Case IV) were observed by SW‐AF.E–HffERG test on the four patients to analyze the light‐ and dark‐adapted vision.IffERG profiling of a healthy individual was obtained as the reference. Data information: In E–I, Y‐Axis: microvolts; X‐Axis: milliseconds.

Four patients of β‐thalassemia intermedia with a history of blood transfusion were subject to iron chelation by DFO for at least 16 years (Table 1). These patients visited the clinic due to visual impairment. Unlike typical signs of iron deposition in the eye, such as corneal iron lines, lens changes, etc., color fundoscopy showed a spectrum of distinctive pathologies, including mottling (Fig 1A), lack of intraretinal pigmentation (Fig 1A and B), diffuse depigmentation (Fig 1A–C), multiple atrophic patches of RPE in the macula and peripapillary areas (Fig 1C and D), as well as choroidal sclerotic vessels (Fig 1D). All these pathological abnormalities were evidence of predominant outer retina/RPE damages and prompted us to determine RPE pathology in initiating and progressing DFO‐related degenerative retinopathy.

**Table 1:** Summary of patients' clinical profiles.

Case Number	Age	Gender	BCVA (OD/OS) at first visit	Duration of chelation therapy	Diagnosis	Intake of AKG
I	53	Male	20/80; 20/80	33 years	β‐thalassemia intermedia	No
II	26	Male	20/30; 20/630	17 years	β‐thalassemia intermedia	No
III	53	Male	CF; 20/630	17 years	β‐thalassemia intermedia	No
IV	64	Male	20/150; HM	16 years	β‐thalassemia intermedia	Yes

BCVA, best corrected visual acuity; CF, counting fingers; HM, hand motion.

**Figure EV1:**
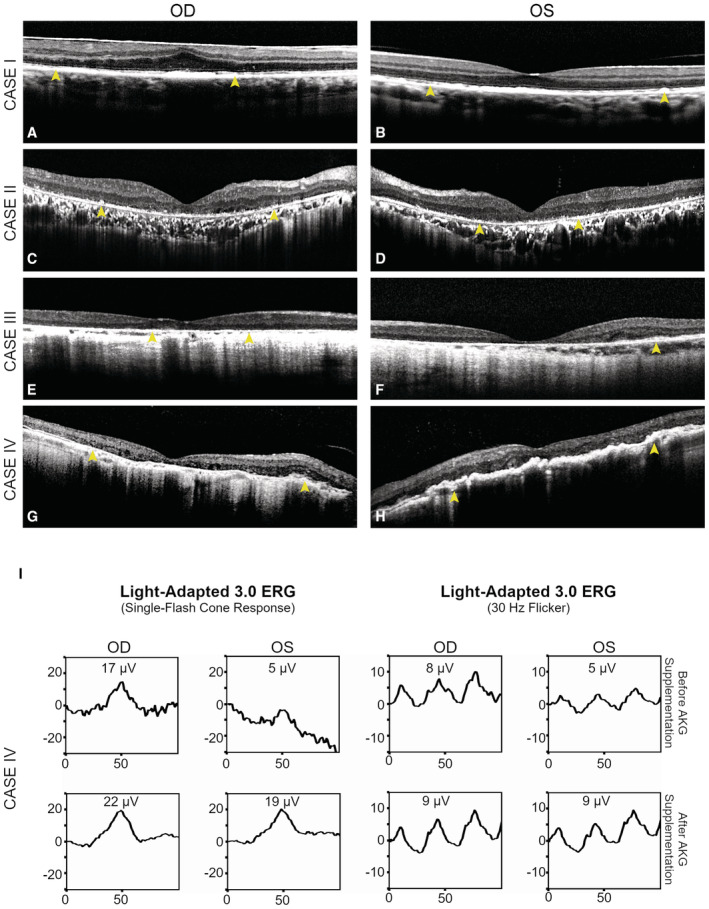
The chelation‐dependent thalassemia patients show retinal degeneration and functional improvement by taking AKG A–HSD‐OCT of the four thalassemia patients with a history of taking DFO showed interruption of the ellipsoid zone (Case I; Gelman *et al*, 2014), decreased photoreceptor nuclear layer, and thinning of the retinal layers (Cases II–IV). Focal thickening and bumps of RPE were also noted in Case I. Granular hyper‐reflective deposits within the RPE (yellow arrows) can be seen in all four cases. An intraretinal degenerative cyst (Case IV) and multiple areas of choroidal hyper‐transmission were also noted in Cases II–IV.IThe ffERG examination on Case IV with continuous supplementation of AKG for 18 months (2 g/day). Repeated ffERG examination (lower panel) on both eyes showed increased amplitudes in light‐adapted single‐flash cone and 30 Hz flicker responses compared with the amplitude prior to taking AKG (upper panel). The exact numbers of the peak value are indicated inside the panel boxes. Y‐Axis: microvolts; X‐Axis: milliseconds.

Short‐wavelength fundus autofluorescence (SW‐AF) imaging was performed to assess potential RPE malfunction due to its capability of capturing the autofluorescence of bisretinoid, which constitutes lipofuscin deposits inside RPE cells. Normal SW‐AF shows homogeneous autofluorescence of RPE with a gradual decline in intensity toward the foveola due to high lutein pigment in the foveal region. Foci of hyper‐ and hypo‐autofluorescence, major signs of morbid or atrophic RPE, respectively (Sparrow *et al*, 2012; Pole & Ameri, 2021), can be seen in SW‐AF images of the patients with DFO retinopathy. Case I showed stippled hyper‐autofluorescence mainly in the macular region without obvious hypo‐autofluorescence, indicating an RPE injury at the early stage of DFO retinopathy (Fig 1A), whereas Cases II, III and IV displayed multiple areas of RPE loss as indicated by diffuse hypo‐autofluorescence (Fig 1B–D). The lesions in the neuroretina were further validated by spectral domain optical coherence tomography (SD‐OCT). Case I exhibited a fragmented ellipsoid zone (EZ) linked to an early stage of DFO‐related retinopathy, granular hyper‐reflective deposits localized to the RPE layer, and thinning of the outer retina (Fig EV1A and B). Case II presented with progressive thinning of the outer nuclear layer and subsidence of the outer plexiform layer with indistinguishable EZ band and external limiting membrane (Fig EV1C and D). Additionally, increased transmission of signals into the choroidal and scleral layers due to extensive RPE atrophy became pronounced in Cases II–IV, which are related to the later stage of degenerative retinopathy (Fig EV1C–H). The image profiling of the four patients, from Case I to IV, delineated the progression of DFO‐related retinopathy that is characterized by initial pathology of RPE prior to the secondary photoreceptor damage.

Visual function assessment by full‐field electroretinography (ffERG) further validated our assessment of disease progression of DFO retinopathy, a typical rod‐cone dysfunction involved in a majority of RPE dystrophies, in the four individual patients. The ffERG responses of Case I appeared normal due to an early stage of disease (Fig 1E), whereas a gradual decline in both dark‐ and light‐adapted responses can be seen in the remaining three patients with advanced DFO retinopathy in comparison with a healthy individual (Fig 1F–I). Closer inspection indicated a minor reduction in the amplitudes of dark‐adapted rod responses and maximal responses in both eyes of Case II (Fig 1F). Significantly reduced amplitudes of the same responses were noted in both eyes of Cases III and IV (Fig 1G and H). The light‐adapted single‐flash responses and 30 Hz flicker showed a gradual deterioration from Case II to IV as decreased amplitude and delayed implicit time became more and more pronounced (Fig 1F–H), which suggested a great severity and extensive secondary photoreceptor degeneration. The ffERG measurement corroborated the disease severity of the four patients assessed by the aforementioned retinal imaging. Remarkably, Case IV had a history of taking AKG (2 g/day) as a supplement for 18 months. The patient reported slight enhancement in visual function since the start of AKG supplementation. Dilated fundus examination and multimodal imaging showed no distinguishable progression compared with his earlier fundus examinations shown in Fig 1D. In a retrospective comparison before and after AKG intake, ffERG revealed pan‐retinal functional improvement in both light‐adapted single‐flash cone response (OD: 17–22 μV; OS: 5–19 μV) and 30 Hz flicker response (OD: 8–9 μV; OS: 5–9 μV) after continuous AKG supplementation (Fig EV1I). Despite RPE as the primary target for DFO, this suggests that the secondary photoreceptor malfunction or degeneration might be halted by AKG supplementation.

**Figure 2:**
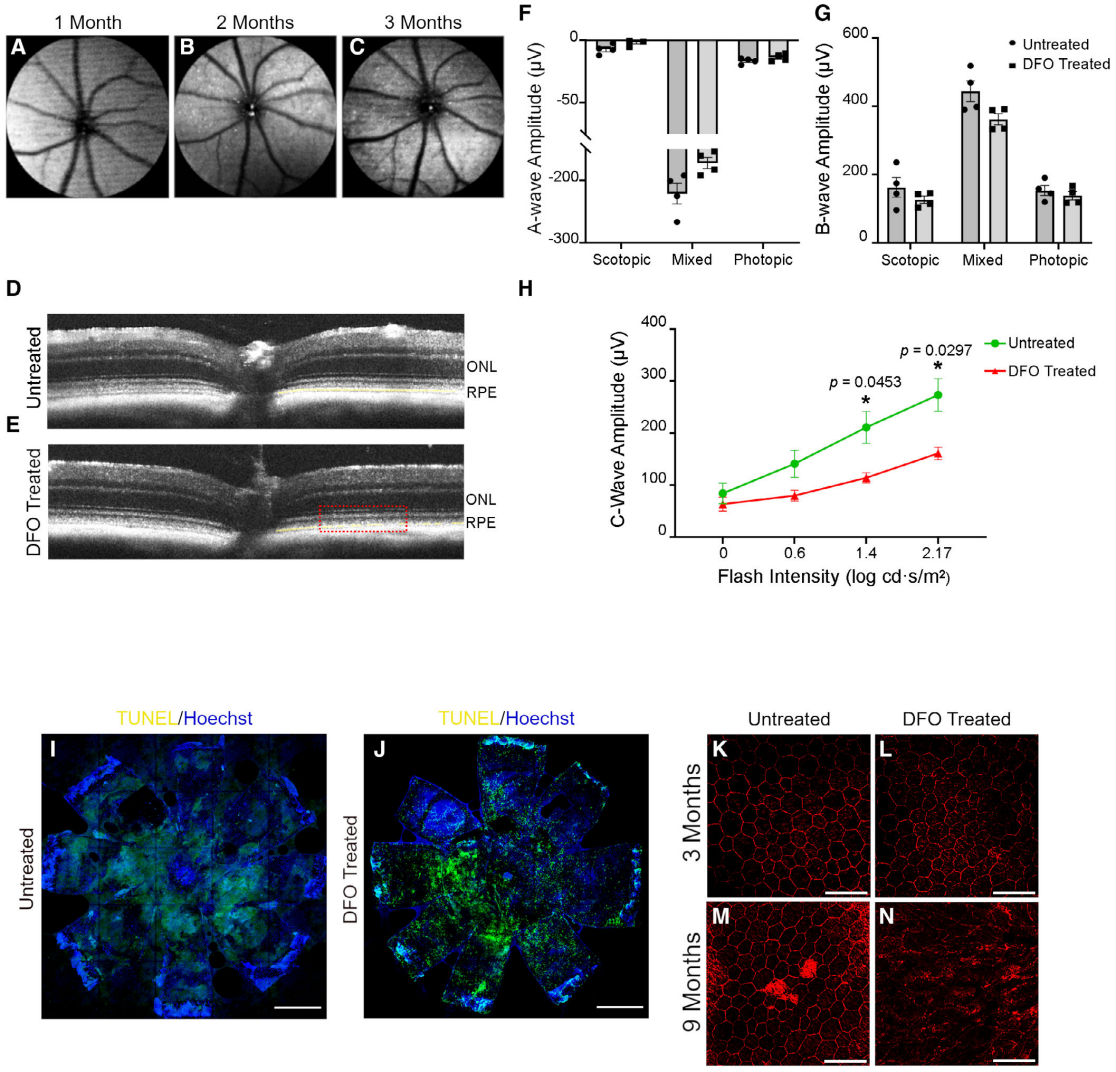
RPE is prone to DFO's toxic effect *in vivo* as a cause of degenerative retinopathy A–CAdult C57BL/6J mice (four months old) were intraperitoneally injected with DFO at 100 mg/kg three times a week for three months in a row. SW‐AF images were acquired longitudinally at one, two and three months post injection.D, ESD‐OCT was performed on seven‐month‐old mice subject to six months of DFO injection. Age‐matched mice without DFO injection were included as the control. Rectangle indicates outer retina/RPE region. Yellow dashed line indicates the RPE layer. ONL: outer nuclear layer; RPE: retinal pigment epithelium.F, GERG was carried out on the mice injected with DFO for six months. Photoreceptor scotopic response was measured under the condition of −3 log cd·s/m^2^; the mixed response was measured under the condition of 0.417 log cd·s/m^2^; the photopic response was measured under the condition of 1.48 log cd·s/m^2^. The statistics are analyzed by unpaired Student's *t*‐test. The results are presented as mean ± S.E.M., *n* = 4 mice for each group.HRPE light response was determined by exposing mouse eyes to a series of flash intensities. Age‐matched untreated mice were included as the controls. The statistics under the same flash intensity are analyzed by unpaired Student's *t*‐test. The results are presented as mean ± S.E.M., *n* = 4 mice for each group. *P < 0.05.I, JFlat mount RPE was harvested from the mice treated with DFO for five months and stained by TUNEL to detect cell death. Hoechst was used for nuclear staining. Untreated mice of similar age were used as the healthy controls. Flat mount images were captured by 10× confocal microscopy and stitched together. Scale bar: 1 mm.K–NThe RPE was dissected from the mice injected with DFO for three and nine months, respectively, and stained with phalloidin. Age‐matched untreated mice were included as the controls. The images were taken at 40× magnification. Scale bar: 50 μm.

### Mouse modeling and *in vitro* characterization of DFO toxicity in RPE

**Figure EV2:**
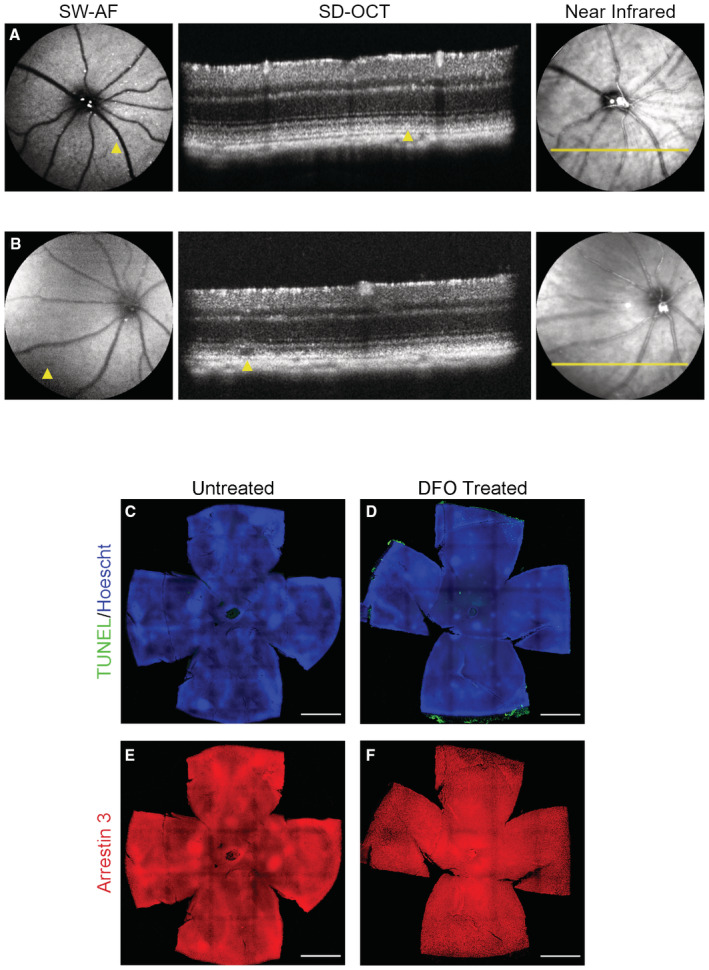
The toxic effects of DFO in the outer retina/RPE area A, BSD‐OCT was performed on DFO‐treated mouse eyes to display potential changes to RPE and retina, respectively. Yellow arrows denote hyper‐reflective signal and outer retinal lesion in SW‐AF and SD‐OCT, respectively (A); and hypo‐reflective signal and lesions located in the photoreceptor outer segment/RPE microvilli area (B). Yellow lines in the near‐infrared images indicate the sections scanned by SD‐OCT.C, DCell death was examined by TUNEL assay in the retinas collected from the mice with DFO treatment for five months.E, FCone photoreceptors were stained by Arrestin 3 in retinal flat mounts from the mice with DFO treatment for five months. Data information: (C–F) The images were captured at 10× magnification and stitched together. Scale bar: 1 mm.

To better understand the ophthalmic toxicity of DFO, we validated the pathological features observed in the clinic by intraperitoneally injecting C57BL/6J wild‐type mice with a dose of 100 mg/kg DFO three times a week from weaning age onwards. Fundus screening by SW‐AF was conducted to identify any changes concomitant with the administration of DFO. We found a progression of the spotting presentation within three months of DFO injection in mice (Fig 2A–C). Hyper‐autofluorescent spots were less notable in the mice injected with DFO for less than one month (Fig 2A). Fundus spotting became pronounced as the administration of DFO continued for two and three months (Fig 2B and C), suggesting accumulation of toxic fluorophores in the RPE. Sectional scanning of DFO‐injected mouse eyes by SD‐OCT distinctly revealed loss of lamination in the outer retina and fragmentation of RPE compared with untreated controls, while photoreceptors and inner retina remained integral (Fig 2D and E). Furthermore, lesions in the outer retina correlate with the hyper‐reflective fluorescence detected by SW‐AF (Fig EV2A). Hypo‐reflective signals were detected that mimic atrophic lesions of the outer retina in the patients with DFO‐related retinopathy, implying an advanced stage of DFO pathology (Fig EV2B). In addition to anatomical changes, we characterized functional impairment of both neuroretina and RPE by ERG. The injection of DFO was extended for six months to better induce functional alterations in the mice. Recordings of both a‐ and b‐waves showed no major difference between DFO‐treated mice and their untreated counterparts, which suggests preservation of retinal functionality (Fig 2F and G). Light responsiveness of RPE, determined by c‐wave (Scholl & Zrenner, 2000), showed a significant decline in mice with DFO intake (Fig 2H). Our data display anatomical and functional damages to RPE that precede neuroretinal degeneration due to DFO intake.

To better delineate differential susceptibility to the impact of DFO between the RPE and the neuroretina, as well as the progression of likely lesions, we collected retina and RPE separately from the mice treated with DFO for three and nine months. Mosaics of retinal flat mount images showed scarce TUNEL‐positive cells in DFO‐treated and control mice (Fig EV2C and D), as well as an approximate number of the (cone) photoreceptor population, indicated by Arrestin 3 (Fig EV2E and F). However, the RPE from DFO‐treated mice showed abundant TUNEL signals compared with untreated controls (Fig 2I and J). It is worth noting that morphological deformation, such as a lack of cell integrity and less homogeneous distribution, can be seen in the mouse RPE sheet that was subject to DFO treatment for three months (Fig 2K and L). Such damage was worsened as the DFO treatment persisted. The hexagonality of individual RPE cells was severely disrupted, losing cell–cell contact between neighboring cells (Fig 2M and N). This mouse modeling of the toxic effect of DFO is in line with our clinical observations: RPE is primarily susceptible to DFO toxicity, which potentially plays a pivotal role in mediating the progression of DFO‐related retinopathy.

**Figure 3:**
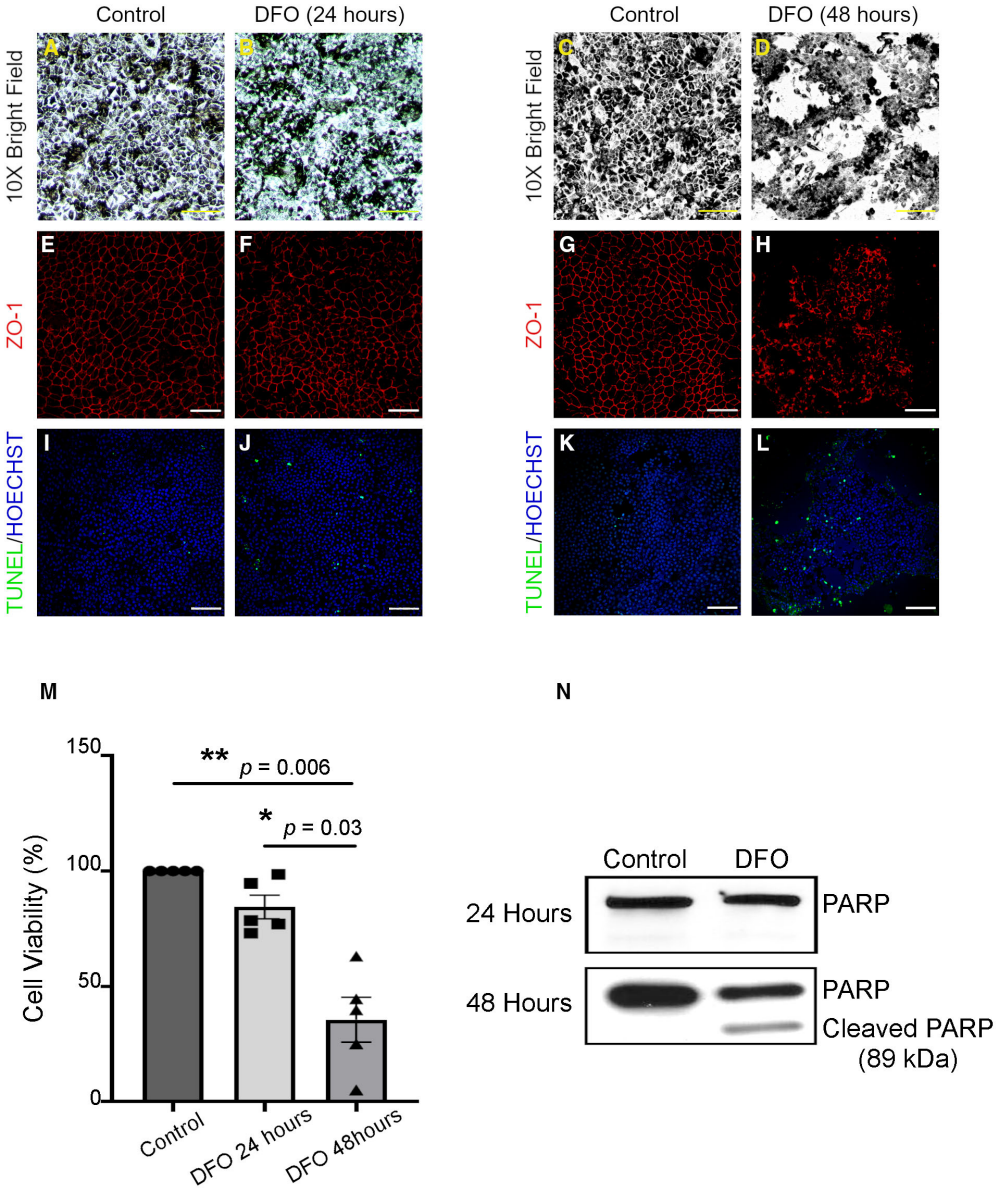
DFO disrupts iRPE integrity *in vitro* iRPE cells were treated with DFO at 100 mM and characterized *in vitro*.
A–DLight microscopic examination of the iRPE cells 24‐ and 48‐h post treatment. Scale bar: 50 μm.E–HiRPE cells were stained with ZO‐1 24‐ and 48‐h post treatment. Untreated iRPE cells were used as the control. Scale bar: 30 μm.I–LCell death among the iRPE population was examined by TUNEL assay 24‐h and 48‐h post treatment. Untreated iRPE cells were included as the control. The cells were counterstained with Hoechst. Scale bar: 30 μm.MCell viability was determined with the iRPE cells subject to DFO treatment for 24‐ and 48‐h. The colorimetric values of the treatment groups were normalized to the control group. The statistics are analyzed by one‐way ANOVA with the Tukey test. The results are presented as mean ± S.E.M., *n* = 5 iRPE lines for each group. *P < 0.05; **P < 0.01.NiRPE cells were collected and lysed for immunoblotting assay against PARP, which was predicted to be 100 kDa. The molecular weight of cleaved PARP was predicted to be 89 kDa. Source data are available online for this figure.

**Figure EV3:**
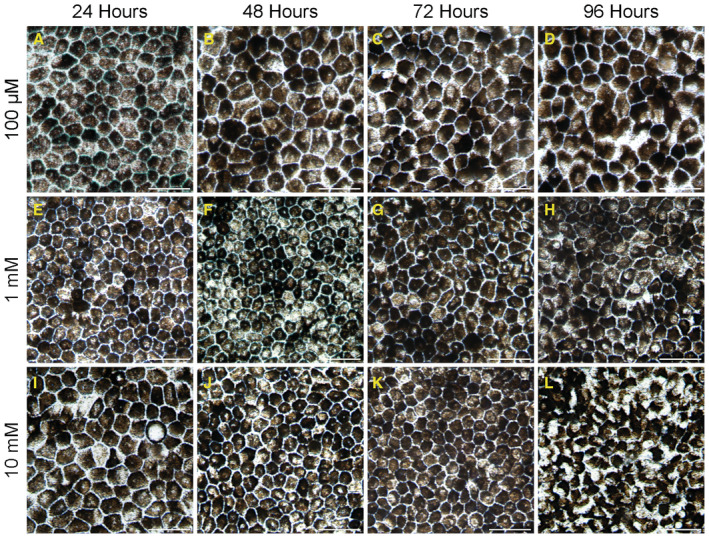
Examination of the toxicity gradient of DFO in iRPE cells A–DThe iRPE cells were treated with DFO for different time points at 100 μM.E–HThe iRPE cells were treated with DFO for different time points at 1 mM.I–LThe iRPE cells were treated with DFO for different time points at 10 mM. Data information: The toxic effect was monitored by light microscopy over a course of time for up to 96‐h until the disruption of cell integrity distinctly appeared. Scale bar: 20 μm. Source data are available online for this figure.

To specify a potential impact of RPE pathology in response to DFO, we obtained iPSC‐derived RPE cells (iRPE) from healthy donors in the clinic and treated the cells with DFO in stepwise concentrations. Our results found a considerable morphological resilience of iRPE cells to lower concentrations of DFO: structural integrity can be maintained without palpable abnormalities in response to DFO at 100 μM and 1 mM for up to 96‐h (Fig EV3A–H); the toxic effect of DFO at 10 mM is only visible 96‐h post treatment (Fig EV3I–L). When the concentration of DFO was increased to 100 mM, despite minimal alteration in the gross morphology of iRPE 24‐h post treatment (Fig 3A and B), the monolayer of iRPE began to lose uniformity and became fragmented 48 h after DFO treatment (Fig 3C and D). Furthermore, ZO‐1 staining revealed dissolution of cell adhesion and perturbation of the hexagonality of DFO‐treated iRPE cells, which is reminiscent of the RPE flat mount from the DFO‐treated mouse as shown in Figs 2 and 3E–H. In parallel, iRPE cell death was examined by TUNEL staining. Compared with the 24‐h time point featuring sporadic TUNEL signal, iRPE cell death was significantly detectable 48‐h post DFO treatment (Fig 3I–L). Furthermore, iRPE cell viability was determined by MTT assay. As anticipated, despite a minimal loss of viable iRPE cells for 24‐h post DFO treatment, there was a significant reduction in viable iRPE cells that were exposed to DFO treatment for 48‐h (Fig 3M). Ongoing iRPE cell death was further verified by examining PARP‐1 due to its profound implication in sensing cell stress and apoptosis. The immunoblot revealed cleavage of PARP‐1 in iRPE cells 48‐h after the DFO treatment (Fig 3N), which concurred with massive cell death and immense stress on iRPE cells resulting from DFO toxicity. Therefore, *in vitro* characterization of atrophic iRPE substantiated our hypothesis about the susceptibility of RPE to DFO toxicity. Of note, DFO at 100 mM was employed for the subsequent assays due to its sufficiency in inducing RPE phenotypes *in vitro*.

**Figure 4:**
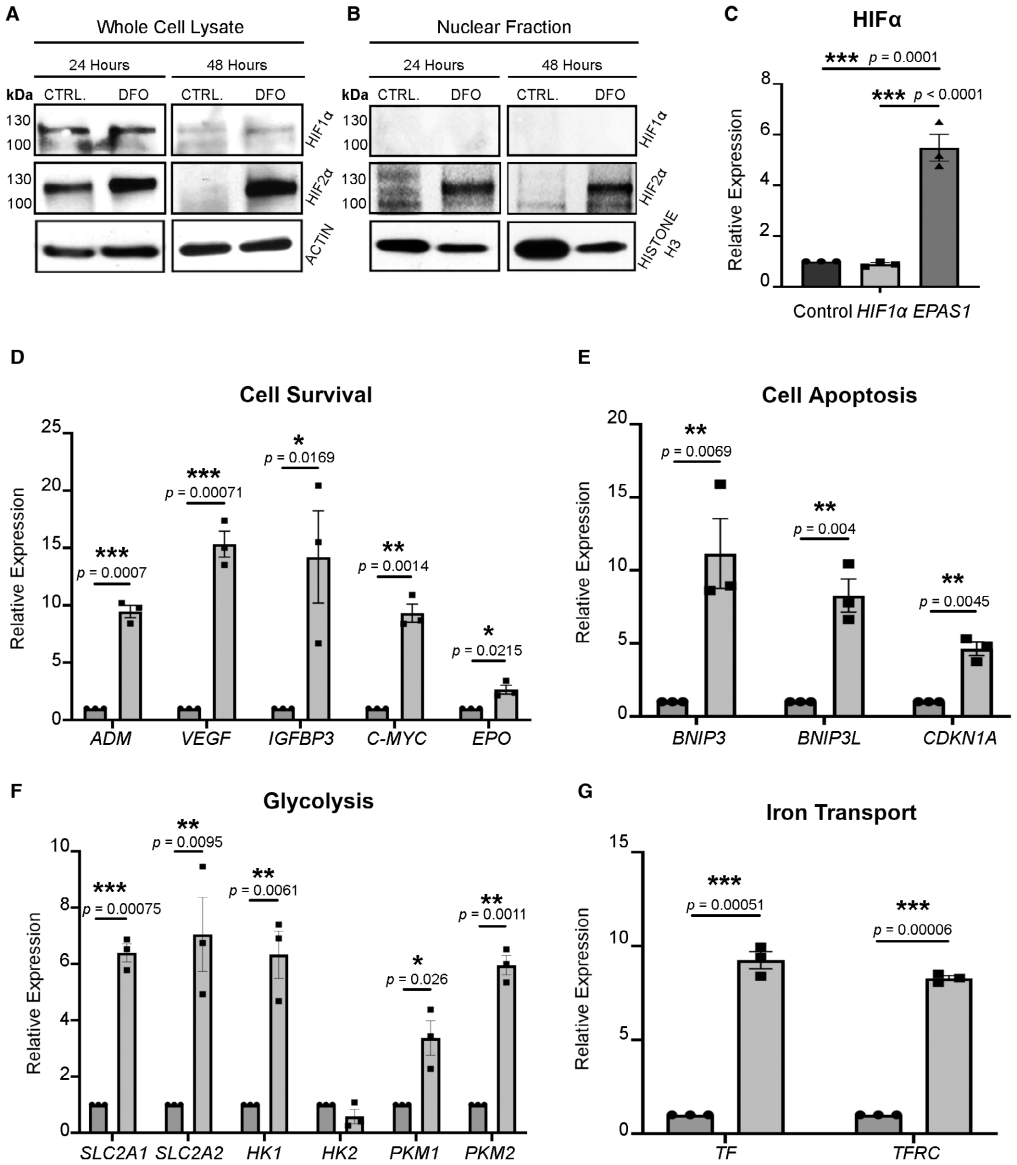
DFO upregulates HIF2α and perturbs its downstream targets AiRPE cells treated with DFO were collected and lysed to measure the expression of HIF1α and HIF2α 24‐ and 48‐h after the treatment. The iRPE cells supplemented with 0.1% DMSO in the culture media were used as the control. Actin was used as the loading control. HIF1α (post‐translationally modified) was predicted to be ~ 100 kDa; HIF2α (post translationally modified) was predicted to be ~ 120 kDa.BiRPE cells treated with DFO were lysed and fractionated by centrifugation to measure the expression of HIF1α and HIF2α in the nuclei 24‐ and 48‐h after the treatment. Untreated iRPE cells were used as the normal control. Histone H3 was used as the loading control.C–GThe transcripts of HIFα‐regulated genes were determined by qPCR with iRPE cells subject to DFO treatment for 24‐h as the template. The cDNA transcript extracted from untreated iRPE was included as the control. The expression of each transcript was normalized to *ACTB* as the housekeeping gene. (C) Measurement of the HIF1α and HIF2α transcripts. The statistics are analyzed by one‐way ANOVA with the Tukey test. The results are presented as mean ± S.E.M., *n* = 3 iRPE lines for each group. ***P < 0.001. (D) Measurement of cell‐survival‐related transcripts regulated by HIFα. (E) Measurement of apoptosis‐related transcript regulated by HIFα. (F) Measurement of glycolysis‐related transcript regulated by HIFα. (G) Measurement of iron‐transport‐related transcript regulated by HIFα. Data information: (D–G) The statistics are analyzed by ratio paired Student's *t*‐test. The results are presented as mean ± S.E.M., *n* = 3 iRPE lines for each group. *P < 0.05; **P < 0.01; ***P < 0.001. Round dots: untreated iRPE; square dots: DFO‐treated iRPE. Source data are available online for this figure.

### Determination of the impact of the HIFα pathway on RPE pathology

The phenotypic characterizations prompted us to investigate the molecular mechanisms underlying the RPE lesions linked to DFO. Iron chelation by DFO dampens PHD, a key enzyme in mediating HIFα degradation in the presence of iron. We therefore hypothesized that disruption of the HIFα pathway is implicated in the pathological changes of the RPE in response to DFO. Measurements of HIF1α and HIF2α levels in iRPE whole cell lysate revealed that the expression of HIF1α remained low (Fig 4A). Interestingly, the level of HIF1α remained largely unchanged despite the treatment of DFO. In contrast, HIF2α was noticeably elevated post DFO treatment (Fig 4A). Furthermore, the expression of both HIF1α and HIF2α in the nuclear fraction was assayed since nuclear relocation is a key step for HIFα to function as a transcription factor. As anticipated, an increase of HIF2α was significant inside nuclei compared with the untreated group, especially 48‐h post treatment, which suggested nuclear entry of HIF2α was enhanced. HIF1α, on the other hand, was barely detected inside the nuclei of iRPE cells regardless of DFO treatment (Fig 4B). Thus, the results distinctly indicate that HIF2α, instead of HIF1α, is susceptible to the impact of DFO and is more likely to play a major role in affecting RPE pathology.

Since both HIF1α and HIF2α function as transcription factors, measurement of their target genes will shed light on the biological processes involved in the RPE pathology due to exposure to DFO. Real‐time qPCR was performed using iRPE extract as the template. It is worth noting that the transcript of *EPAS1* (HIF2α) is increased to a greater extent than that of *HIF1A* (HIF1α), which remained stable despite the presence of DFO (Fig 4C). This result confirmed that *EPAS1* is hyperactivated in response to DFO and convinced us of a major role of HIF2α, in lieu of HIF1α, underlying the RPE damage.

**Figure EV4:**
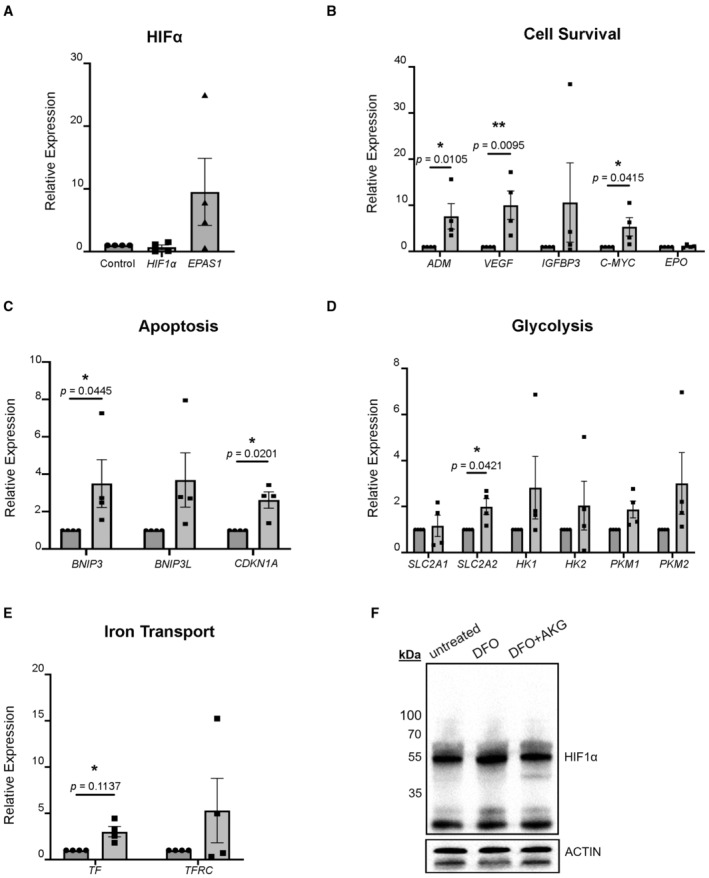
Measurement of HIFα and its target genes in response to DFO in iRPE cells A–EThe HIFα‐regulated transcripts were determined by qPCR with iRPE cells subject to DFO treatment for 48‐h. The cDNA transcript extracted from the untreated iRPE was included as the control. The level of each transcript was normalized to *ACTB*. (A) Measurement of the HIF1α and HIF2α transcripts. The statistics are analyzed by one‐way ANOVA with the Tukey test. The results are presented as mean ± S.E.M., *n* = 4 iRPE lines for each group. (B) Measurement of cell‐survival‐related transcripts regulated by HIFα. (C) Measurement of apoptosis‐related transcript regulated by HIFα. (D) Measurement of glycolysis‐related transcript regulated by HIFα. (E) Measurement of iron‐transport‐related transcript regulated by HIFα.FRPE lysate from the one‐year‐old mice subject to 10‐month DFO treatment with or without concomitant supplementation of AKG was used for immunoblotting against HIF1α. The RPE harvested from the age‐matched untreated mice was included as the control. HIF1α (degraded) was predicted to be 40–80 kDa. Actin was used as the loading control. Data information: (B–E) The statistics are analyzed by ratio paired Student's *t*‐test. The results are presented as mean ± S.E.M., *n* = 4 iRPE lines for each group. *P < 0.05; **P < 0.01. Round dots: untreated iRPE; square dots: DFO‐treated iRPE. Source data are available online for this figure.

Meanwhile, we measured the expression of target genes of HIF2α that are implicated in multiple pathophysiological processes in RPE cells. Firstly, a panel of genes linked to cell survival (*ADM*, *MYC*, *IGFBP3*, *VEGF* and *EPO*) and cell death (*BNIP3*, *BNIP3L* and *CDKN1A*) were tested, all of which were significantly increased (Fig 4D and E) as early as 24‐h post DFO treatment. This provides strong evidence of compromised RPE cell viability. Considering the central role of HIFα in glycolytic regulation (Formenti *et al*, 2010), a bulk of genes pertaining to glycolysis, such as *SLC2A1* (GLUT1), *SLC2A2* (GLUT2), *HK1*, *HK2*, *PKM1* and *PKM2* were also tested. All these genes except *HK2*, a minor regulator in the RPE (Cheng *et al*, 2021), were significantly increased, which suggested a profound involvement of glycolysis in the RPE cells upon the treatment of DFO (Fig 4F). As the most iRPE cells became visibly moribund 48‐h after treatment, the majority of the downstream genes became less responsive toward DFO in comparison to their untreated counterparts (Fig EV4A–E). Based on the measurement of the transcript of HIFα target genes, we concluded that HIF2α serves as a vital player in dictating RPE cell response to the toxic effect of DFO.

### DFO disrupts iron homeostasis and shifts the metabolic paradigm in iRPE cells

**Figure 5:**
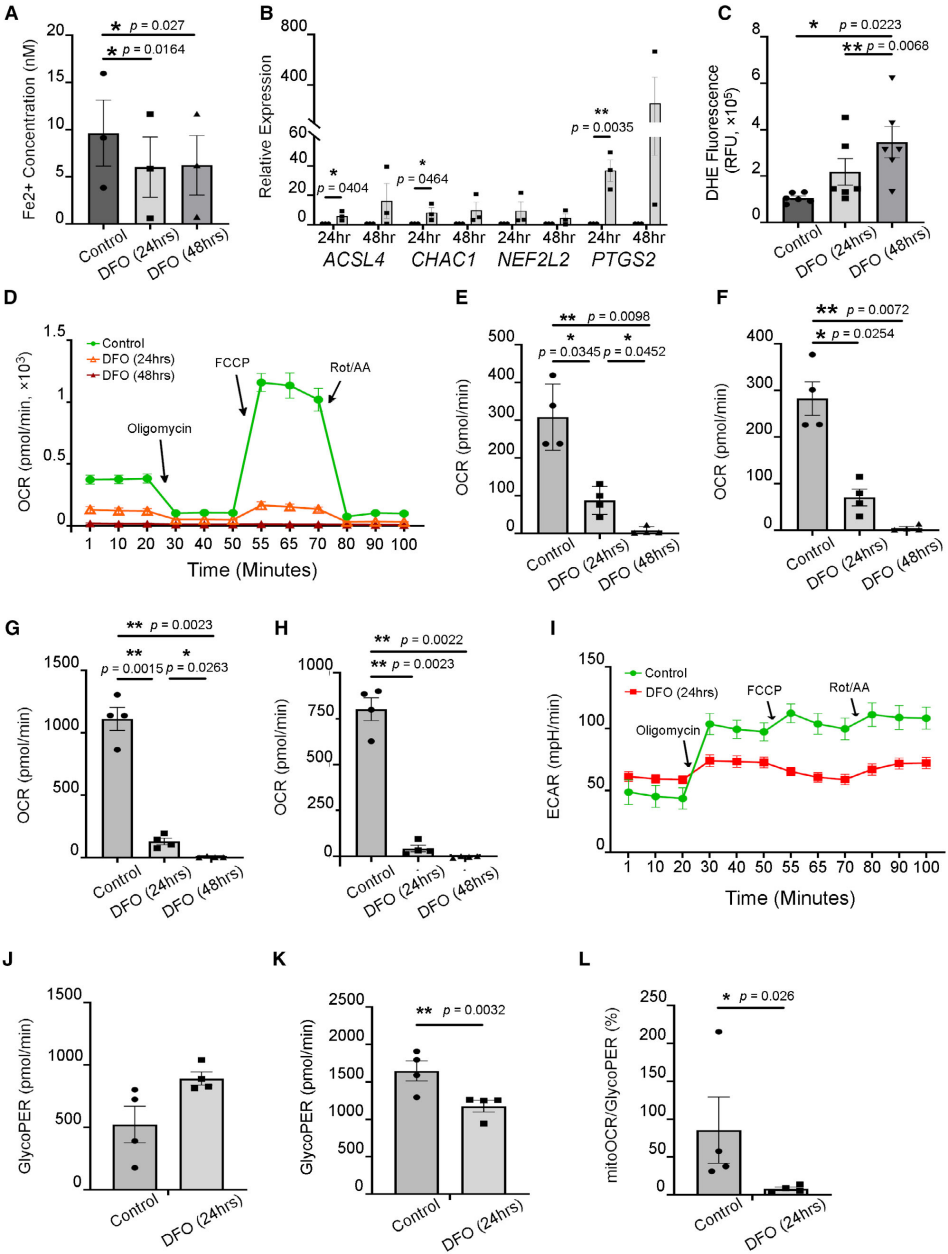
DFO perturbs iron homeostasis and causes metabolic disruption in iRPE cells The iRPE cells were treated with DFO at 100 mM for 24‐ and 48‐h.
AFerrous iron was measured. The statistics are analyzed by one‐way ANOVA with the Tukey test. The results are presented as mean ± S.E.M., *n* = 3 iRPE lines for each group. *P < 0.05.BThe transcripts of ferroptosis‐associated markers were determined by qPCR using iRPE cells as the template. The RNA extract from untreated iRPE was included as the control. The level of each transcript was normalized to *ACTB*. The statistics are analyzed by ratio paired Student's *t*‐test for each target gene. The results are presented as mean ± S.E.M., *n* = 3 iRPE lines for each group. *P < 0.05; **P < 0.01. Round dots: untreated iRPE; square dots: DFO‐treated iRPE.CThe level of ROS was measured. The statistics are analyzed by one‐way ANOVA with the Tukey test. The results are presented as mean ± S.E.M., *n* = 6 iRPE lines for each group. *P < 0.05, **P < 0.01. DHE: Dihydroethidium.DMitochondrial respiration of the iRPE cells with DFO treatment was determined by seahorse extracellular flux assay. OCR was determined over a course of time. Each data point is shown as mean ± S.E.M., *n* = 4 iRPE lines for each group. OCR: oxygen consumption rate; FCCP: carbonyl cyanide 4‐(trifluoromethoxy) phenylhydrazone; Rot: rotenone; AA: antimycin A.E–HBasal respiration (E), ATP production (F), maximal respiration (G) and spare mitochondrial capacity (H) were calculated based on OCR tracing readout. The statistics are analyzed by one‐way ANOVA with the Tukey test. The results are presented as mean ± S.E.M., *n* = 4 iRPE lines for each group. *P < 0.05, **P < 0.01.IGlycolytic stress of the iRPE cells in the presence of DFO was determined by Seahorse extracellular flux assay. ECAR was determined over a course of time. Each data point is shown as mean ± S.E.M., *n* = 4 iRPE lines for each group. ECAR: extracellular acidification rate.J–LBasal glycolysis (J), compensatory glycolysis (K) and the ratio between mitochondrial OCR and glycolysis (L) were determined based on the ECAR tracing readout. The results are presented as mean ± S.E.M., *n* = 4 iRPE lines for each group. *P < 0.05, **P < 0.01.

Next, we sought to reveal the molecular basis underlying RPE cell death caused by DFO toxicity. Firstly, we continued to explore the impact of hyperactive HIF2α on iron homeostasis. *TF* and *TFRC*, two HIFα‐target genes implicated in iron transport, were measured. Both genes were increased, hinting at increased demand for iron transport in response to depletion (Fig 4G). Meanwhile, the level of intracellular Fe^2+^ was measured to determine the potency of chelating ferrous iron by DFO. Fe^2+^ underwent a significant loss 24‐h post DFO treatment (Fig 5A), which indicates a iron deficiency. Interestingly, both TF and TfR are considered major regulators of ferroptosis, a unique type of cell death (Chen *et al*, 2020; Feng *et al*, 2020). Significant increases of both molecules prompted us to test whether ferroptosis was implicated in DFO‐related RPE cell death. By testing several established markers for ferroptosis, including *ACSL4*, *CHAC1*, *NEF2L2* and *PTGS2* (Chen *et al*, 2021), we noted that all the targets except *NEF2L2* were significantly upregulated 24‐h after DFO treatment, which strongly suggests a propensity to ferroptosis in atrophic RPE due to DFO toxicity (Fig 5B).

Considering the involvement of iron in ROS production, we further clarified a likely change related to production of ROS inside the iRPE cells. Strikingly, the level of ROS was increased 24 h post DFO treatment and continued to worsen over time (Fig 5C). In order to obtain molecular insights from a metabolic perspective, we hypothesized that aberrant mitochondrial function underlies the RPE pathological changes. The seahorse extracellular flux assays were performed. Based on our data, an overall suppression of mitochondrial function in DFO‐treated iRPE cells is distinct (Fig 5D): both basal and maximal respiration as well as the spare respiratory capacity of mitochondria declined significantly in iRPE cells in the presence of DFO that is explicable to the reduction in ATP production (Fig 5E–H). Moreover, we analyzed glycolysis to better delineate metabolic perturbation linked to DFO treatment in the iRPE cells. Our results revealed a relatively complex picture (Fig 5I): on the one hand, basal glycolysis appears to raise in response to DFO treatment despite lack of statistical significance (Fig 5J); on the other hand, the compensatory glycolysis is significantly reduced (Fig 5K). More strikingly, the ratio of basal mitochondrial respiration over glycolysis is greatly reduced, which strongly hinted at a metabolic shift from oxidative phosphorylation (OXPHOS) to glycolysis (Fig 5L). More importantly, it revealed an overall depletion of energy metabolism as both reserved respiration and compensatory glycolysis are distinctly undermined. To summarize, these results state that iron chelation by DFO disrupts iron homeostasis in company with elevating ROS in iRPE cells. More profoundly, it dampens mitochondrial function, which disrupts the metabolic paradigm and accounts for RPE cell death.

### Improving RPE survival by AKG via ameliorating mitochondrial capacity and inhibiting HIF2α

**Figure 6:**
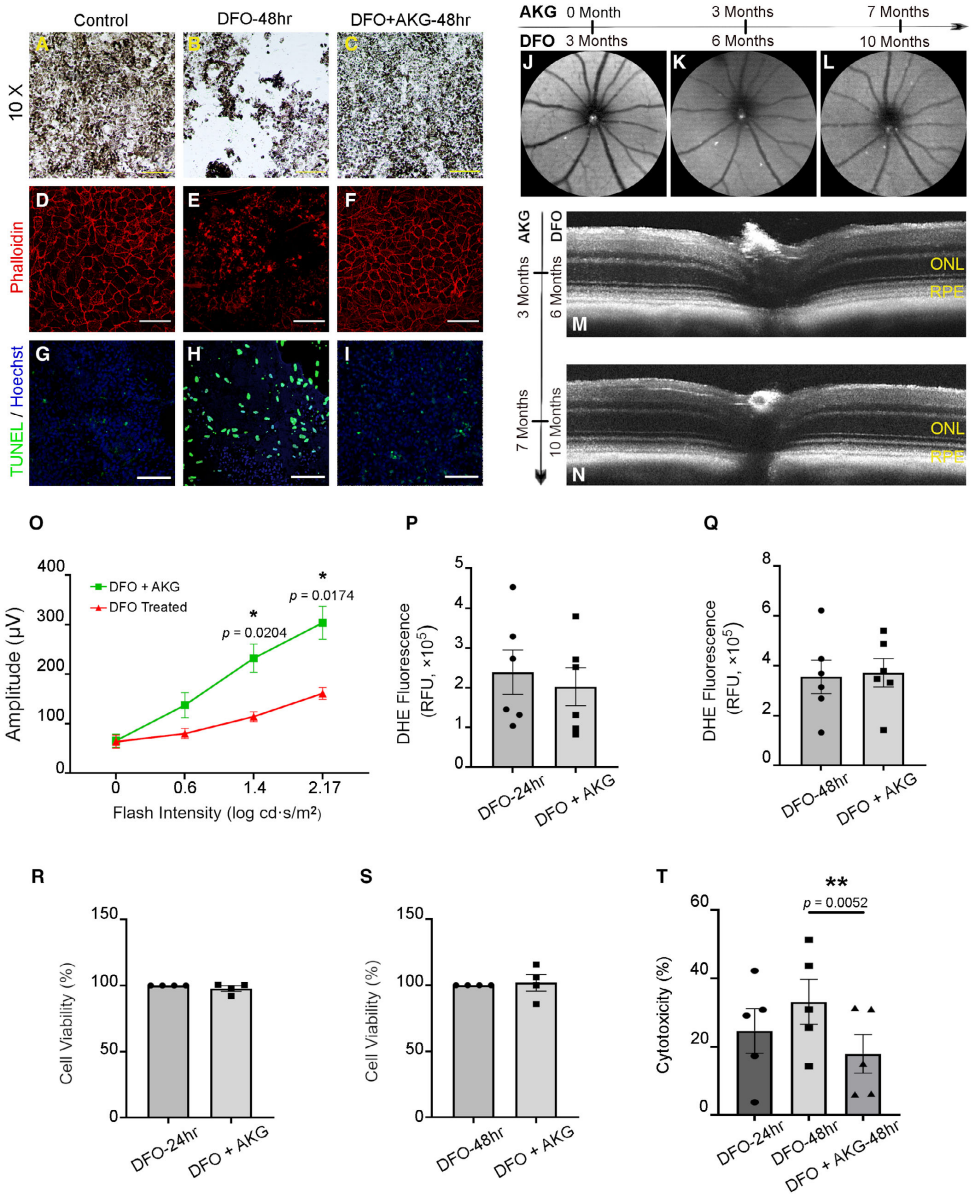
AKG alleviates the RPE damage related to the toxic effect of DFO A–CLight microscopy of morphological protection of AKG against DFO toxicity on iRPE. The cells were treated with DFO at 100 mM for 48 h (B). AKG was supplemented at 10 mM simultaneously (C). The control group was supplemented with 0.1% DMSO (A). Scale bar: 100 μm.D–FThe DFO‐treated iRPE cells with or without AKG supplementation were subject to phalloidin staining (E and F). The iRPE cells treated with 0.1% DMSO‐containing media were included as the controls (D). The images were captured at 40× magnification. Scale bar: 30 μm.G–IDetection of cell death in the DFO‐treated iRPE cells with or without AKG supplementation by TUNEL staining. Hoechst was used for nuclear staining. The images were captured at 40× magnification. Scale bar: 30 μm.J–LSW‐AF was performed longitudinally on the mice treated with DFO for up to 10 months in conjunction with supplementation of AKG for seven months.M, NSD‐OCT was performed on the DFO‐treated mice with AKG supplementation at three and seven months, respectively. ONL: outer nuclear layer; RPE: retinal pigment epithelium.OThe c‐wave ERG was performed on the mice treated by DFO (seven months) with AKG (six months). DFO‐treated mice without AKG supplementation were included as the controls. The statistics associated with each flash intensity are analyzed by unpaired Student's *t*‐test. The results are presented as mean ± S.E.M., *n* = 4 mice for each group. *P < 0.05.P–TThe iRPE cells treated with DFO (100 mM) with or without supplementation of AKG (10 mM) were collected for the following assays: (P, Q) The level of ROS was measured by the dihydroethidium (DHE) fluorometric assay. The statistics are analyzed by paired Student's *t*‐test. The results are presented as mean ± S.E.M., *n* = 6 iRPE lines for each group. (R, S) The cell viability was determined by the MTT assay 24 and 48 h post treatment. The colorimetric values were normalized to the control group. The statistics are analyzed by paired Student's *t*‐test. The results are presented as mean ± S.E.M., *n* = 4 iRPE lines for each group. (T) Cytotoxicity in iRPE cells was tested by the colorimetric LDH cytotoxicity assay. The statistics are analyzed by one‐way ANOVA with the Tukey test. The results are presented as mean ± S.E.M., *n* = 5 iRPE lines for each group. **P < 0.01.

It is essential to address whether DFO's toxic effect on RPE cells can be mitigated by correcting the aberrant metabolic pathways and suppressing the HIF2α signaling. AKG, another indispensable co‐factor for PHD in proteasomal degradation of HIFα and a key intermediary metabolite in the tricarboxylic acid (TCA) cycle, was supplemented in conjunction with DFO. Light microscopy of iRPE cells displayed maintenance of the gross morphology of the DFO‐treated iRPE due to supplementation of AKG (Fig 6A–C). Fluorescence microscopy further characterized preservation of the hexagonal structure of the iRPE cells (Fig 6D–F). Importantly, cell death, examined by TUNEL assay, was ameliorated (Fig 6G–I). Structural characterization of iRPE cells suggested an antagonistic effect of AKG against DFO toxicity. This protective effect was further tested *in vivo* by supplementing DFO‐injected mice with AKG in drinking water. SW‐AF was carried out on these mice in a longitudinal manner in order to monitor the pathological progression. Our examination showed no significant development of mottling fundus in these DFO‐treated mice supplemented with AKG for seven months (Fig 6J–L). SD‐OCT revealed substantial preservation of the outer retina/RPE region, especially the integrity of the RPE due to the presence of AKG in addition to continual DFO injection (Fig 6M and N). Importantly, ERG c‐wave recordings convinced us of the maintenance of RPE functionality as a better response of the RPE to the flash stimuli can be seen (Fig 6O). Moreover, we validated the protective effect of AKG at a cellular level. We failed to detect a significant decrease in ROS production as originally hypothesized (Fig 6P and Q). The cell viability examined by MTT assay was not significantly improved either (Fig 6R and S). The MTT assay is a measurement of mitochondrial reaction by essence. Alternatively, we tested the DFO‐associated cell toxicity by lactate dehydrogenase (LDH) assay, which measures leakage of LDH due to damage to cell integrity. Our data revealed a significantly reduced efflux of LDH linked to AKG supplementation for 48 h (Fig 6T), which indicated likely maintenance of cell integrity. Thus far, we conclude that DFO toxicity can be mitigated by AKG both anatomically and functionally *in vivo*. Since its antagonistic effect against DFO at the cellular level remained elusive, we hypothesized an implication of AKG in metabolic regulation that determines its capability of antagonizing DFO toxicity.

**Figure 7:**
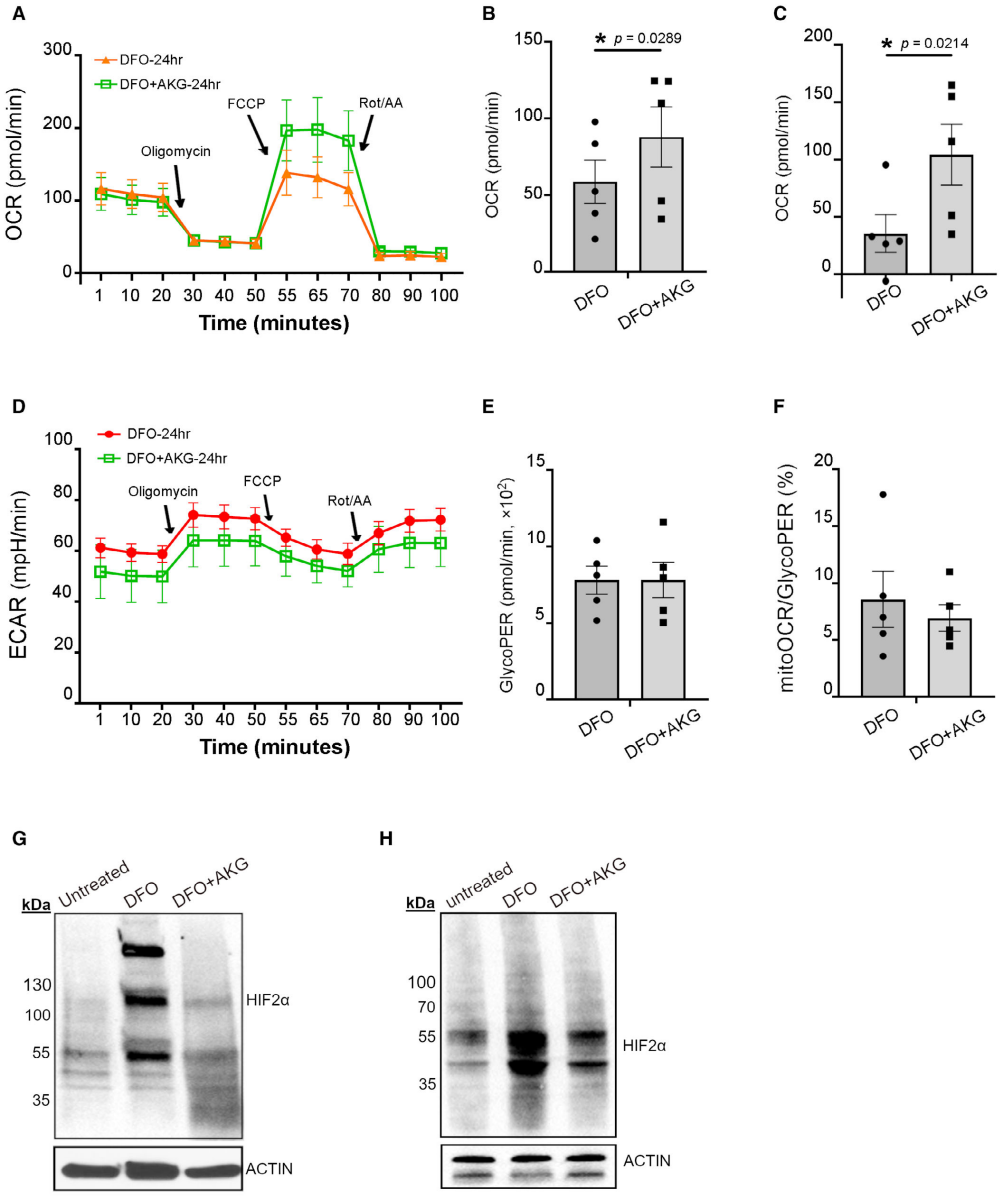
AKG preserves mitochondrial capacity and inhibits HIF2α upregulation in iRPE cells AMitochondrial respiration of the DFO‐treated (100 mM) iRPE cells with AKG supplementation (10 mM) for 24 h was determined by seahorse extracellular flux assay. OCR was measured over a course of time. Each dot is shown as mean ± S.E.M., *n* = 5 iRPE lines for each group. OCR: oxygen consumption rate; FCCP: carbonyl cyanide 4‐(trifluoromethoxy) phenylhydrazone; Rot: rotenone; AA: antimycin A.B, CMaximal (B) and spare capacity (C) of mitochondrial respiration were determined based on the OCR tracing readout. The statistics are analyzed by paired Student's *t*‐test. The results are presented as mean ± S.E.M., *n* = 5 iRPE lines for each group. *P < 0.05.DGlycolytic stress of the DFO‐treated (100 mM) iRPE cells with AKG supplementation (10 mM) for 24 h was determined by seahorse extracellular flux assay. ECAR was measured over a course of time. Each dot is shown as mean ± S.E.M., *n* = 5 iRPE lines for each group. ECAR: extracellular acidification rate.E, FThe basal (E) and the ratio (F) between mitochondrial OCR and glycolysis were determined based on the ECAR tracing readout. The statistics are analyzed by paired Student's *t*‐test. The results are presented as mean ± S.E.M., *n* = 5 iRPE lines for each group.GThe DFO‐treated (100 mM) iRPE cells supplemented with AKG (10 mM) for 48 h were lysed for immunoblotting against HIF2α. The iRPE cells supplemented with 0.1% DMSO in the culture media were used as the controls. HIF2α (post translationally modified) was predicted to be ~120 kDa. Actin was included as the loading control.HRPE lysate from the one‐year‐old mice subject to 10‐month DFO treatment with or without concomitant supplementation of AKG was used for immunoblotting against HIF2α. The RPE of the age‐matched untreated mice was included as the control. HIF2α (degraded) was predicted to be 40–80 kDa. Actin was used as the loading control. Source data are available online for this figure.

In order to address this question, we conducted seahorse extracellular flux assays for recording iRPE metabolic changes in the presence of AKG. The overall tracing of mitochondrial respiration indicated limited potency of AKG in improving mitochondrial function against the damage by DFO: AKG fails to fundamentally augment the basal respiration in iRPE cells that is suppressed by DFO (Fig 7A). However, it increases the maximal mitochondrial respiration and the spare respiratory capacity (Fig 7B and C), which highly implies preservation of reserved mitochondrial capacity in iRPE cells against the damage resulting from DFO toxicity. Despite lack of statistical significance, glycolysis dropped in AKG‐supplemented iRPE cells and there was a likely restoration of the metabolic pathway to OXPHOS (Fig 7D–F). Finally, we sought to answer whether AKG would affect HIF2α in RPE associated with DFO. Immunoblotting of iRPE whole‐cell lysate showed that augmented HIF2α due to DFO treatment was significantly suppressed by co‐treatment of AKG (Fig 7G). In parallel, we examined the inhibitory effect of HIF2α by AKG *in vivo*. Despite a significant degradation of HIF2α due to its volatility to normoxia while tissue dissection, an abundant expression of HIF2α can still be seen in the mice subject to the DFO treatment for 10 months, in comparison to their wild‐type counterparts. The elevation can be distinctly suppressed by supplementing AKG as shown by the western blot (Fig 7H). Likewise, we re‐probed same samples to test HIF1α in response to DFO with and without AKG treatment *in vivo*. As anticipated, a change in its expression is hardly detectable in the mouse RPE (Fig EV4F), which verified inhibition of HIF2α signaling instead of HIF1α by AKG in the RPE. We reasoned that AKG reduces the level of HIF2α and protects the respiratory capacity against the toxic effect of DFO in RPE mitochondria. Our findings provided experimental evidence that thalassemia patients may benefit from the intake of AKG to ameliorate DFO‐related retinopathy.

## Discussion

Iron overload disorders are a group of heterogeneous conditions in clinic resulting from blood transfusion, hemodialysis, dietary consumption or hemochromatosis. They mainly affect metabolically active organs, such as heart, liver, etc., and require close medical attentions (Siah *et al*, 2005; McDowell *et al*, 2022). At the cellular level, iron burden perturbs redox reaction and results in cell death (Dixon & Stockwell, 2014; Masaldan *et al*, 2018). Naturally, iron chelation has been attempted to prevent cell damage, such as ferroptosis, an iron‐dependent type of cell death closely related to ROS production (Dixon *et al*, 2012; Hirschhorn & Stockwell, 2019; Tang *et al*, 2021). Iron overload imposes a remarkable impact on inherited retinopathies and age‐related macular degeneration via inducing oxidative stress (Hahn *et al*, 2003; Dunaief, 2006; Mao *et al*, 2014; Ueda *et al*, 2018). It was reported that an elevated level of iron is identifiable in aging retina in the human population (He *et al*, 2007). Removal of intracellular iron by deferiprone (DFP), another major iron chelator, inhibits ROS and alleviates retinal degeneration in a mouse model of inherited retinopathy (Ueda *et al*, 2018), whereas this study shed light on the consequences of excessive iron loss due to the chelation therapy by DFO, which undermines mitochondrial function and eventually leads to RPE death. It focuses on the other end of the spectrum of iron homeostasis and established a linkage between RPE lesions and iron deficiency. In fact, DFO's pro‐survival impact was recognized even earlier due to its capability of suppressing ferroptosis (Dixon *et al*, 2012). However, its cytotoxicity is scarcely addressed so far. An investigation noted the impact of DFO on inducing cell death at a significantly high dose (Bajbouj *et al*, 2018). This study, among the few, reveals a likely Janus‐faced role of iron removal in cell viability that is associated with the dosage of DFO. Additionally, disparities among the iron chelators should be examined at a subcellular level due to their capabilities in influencing mitochondria: DFP is predicted to be more potent in safeguarding mitochondrial quality than its counterparts (Hara *et al*, 2020). Our study further highlights a clinical model to study iron balance for governing the well‐being of the RPE. It broadens the conceptual scope of iron‐dependent cell viability and raises awareness of the utilization of iron chelators in the clinic. Naturally, unveiling the molecular basis that governs iron homeostasis in RPE and mediates the atrophic lesion will become a key next step.

Mechanistically, we believe that a battery of iron‐dependent biological functions, such as the electron transfer chain, enzymatic reactions, etc. could be perturbed. HIFα was nevertheless underlined in this study due to the profound implication of DFO in activating HIFα. Of note, HIF2α has been established as a critical mediator in iron homeostasis. It incurs a “ferroptosis‐like” cell fate and potentiates cysteine oxidation to elevate ROS (Mastrogiannaki *et al*, 2009; Singhal *et al*, 2021), which corroborates our observation in the DFO‐treated RPE. This study emphasizes critical influence of HIF2α in metabolic reprogramming that has been underestimated in the retina (Majmundar *et al*, 2010; Kurihara *et al*, 2016; Downes *et al*, 2018). Mounting evidence revealed that certain intermediary metabolites of glycolysis/OXPHOS are neuroprotective against oxidative stress (Sawa *et al*, 2017). Replenishing metabolites of the TCA cycle, such as AKG, halts progressive retinal degeneration in the inherited retinopathies (Wert *et al*, 2020; Rowe *et al*, 2021), which is in line with our findings. It is worth noting that the AKG's protection against DFO toxicity in RPE seems limited due to its insufficient correction of mitochondrial deficit and aberrant glycolysis. Instead, it maximally preserves mitochondrial capacity that delays the deterioration of DFO‐related mitochondrial deficit to a great extent. It lays the foundation for clinical management: intake of AKG halts the degenerative retina but may not reverse the course of disease progression. Early preventive measures should be considered to minimize the damage.

RPE was proposed to be a key player in a delicate metabolic ecosystem of energy conversion and consumption in the retina (Kanow *et al*, 2017). It highly depends on mitochondria. The glycolysis‐dynamic RPE could be detrimental to the sensory retina (Zhao *et al*, 2011; Kurihara *et al*, 2016). Evidently, this study shows mitochondrial dysfunction in RPE with aberrant glycolysis that triggers RPE lesions. However, we also noticed inhibition of compensatory glycolysis in RPE cells in response to DFO. It reveals a profound damage to energy metabolism that invariably affects the well‐being of RPE. Previous studies predominantly stressed photoreceptor pathology as a consequence while likely damages to RPE remained unclear. This study probed into the pathological alterations in the RPE, which will be enlightening to RPE‐related retinal degeneration. Next, it is critical to understand the metabolic equilibrium that connects photoreceptors and RPE.

In an early report of ophthalmic presentations in thalassemia patients subject to chelation therapy by DFO, RPE abnormalities had been documented and predicted to be the primary lesion, which is consistent with our postulation (Oh *et al*, 2020). A temporal asynchrony of tissue damage was further validated by our investigation: RPE lesions precede neurosensory damage after initiation of DFO treatment. Interestingly, we noticed that the susceptibility of the RPE to DFO toxicity could be influenced multi‐factorially. The primary function of the RPE as a barrier and scavenger determines its vulnerability to environmental stressors. Moreover, resting potentials of the cell membrane may impose another layer of influence on the penetration of DFO. Human photoreceptors were recorded to be more depolarized than other types of neurons and RPE cells, which presumably impedes cellular uptake of positively charged DFO (Quinn & Miller, 1992; Hu *et al*, 1994; Kawai *et al*, 2001; Porter *et al*, 2005; Kokkinaki *et al*, 2011). Lastly, ATP dependence and cationic drug transporters could be critical to the intracellular transport of DFO to the RPE cells (Han *et al*, 2001).

Clinically, serum ferritin is extensively used to monitor iron level in transfusion‐dependent patients. However, doubts over its sensitivity and specificity make it less sufficient to assess local iron burden. Imaging screening, or even invasive biopsy, is used to inspect iron overload (Anderson *et al*, 2001; Hoffbrand *et al*, 2012; Masini *et al*, 2022). Therefore, regular ophthalmic examinations and the need for reliable markers specific to the eye are necessary. The small cohort of the patients involved in this study is a limitation; although recruiting patients with orphan disorders is always challenging. Therefore, this study relied on iRPE cells derived from healthy donors as well as wild‐type mice. The heterogeneity of genetic compositions of the iRPE in this study is noteworthy. Epistasis, or the presence of genetic modifiers, may remarkably influence our interpretations of the results. Moreover, ophthalmic presentations of DFO‐related retinopathy are mostly associated with transfusion‐dependent thalassemia due to mutated β‐hemoglobin, which is encoded by hemoglobin subunit β (*HBB*). Thus far, more than 350 *HBB* mutations have been associated with thalassemia (Taher *et al*, 2021). Hemoglobinopathies due to the *HBB* mutations encompass a group of clinically distinctive hematological disorders. Diagnosis of β‐thalassemia heavily relies on blood smear and hemoglobin electrophoresis (Kohne, 2011; Origa, 2017). Lacking molecular diagnosis for individual patients hindered us from performing patient‐specific modeling. Additionally, multiple studies pointed out that ocular manifestations were present in less than 10% of the total patients with a history of taking DFO (Olivieri *et al*, 1986; Cohen *et al*, 1990; Baath *et al*, 2008), which appeared to be validated by our mouse modeling and implied complexity of initiation and progression of the RPE damage due to iron chelation. Inevitably, identifying interactions of *HBB* and its variants with iron regulation will be informative. Larger cohorts or isogenic controls are indispensable to address the genetic predisposition of the RPE atrophy to DFO toxic effects, which will collectively facilitate the clinical treatment of ophthalmic disorders associated with iron chelation.

## Materials and Methods

### Phenotypic assessment in the clinic

Four patients of thalassemia intermedia who had a history of taking DFO due to long‐term blood transfusion for various durations were analyzed in this study. The study was approved by the Institutional Review Board (IRB) at Columbia University Medical Center. The de‐identified human data were analyzed according to Protocol AAAR8743 and the consent form of each patient was waived due to the retrospective nature of the study. One patient (Case IV) had taken AKG (2 g/day) for 18 months prior to this study due to progressive visual deterioration. The ophthalmic examinations were performed on all four patients, including SW‐AF (488 nm excitation), SD‐OCT scans (Spectralis HRA/OCT, Heidelberg Engineering, Heidelberg, Germany) and color fundus photography. SW‐AF was acquired by the Spectralis HRA/OCT (55‐degree field) or the Optos 200 Tx (PLC, Dunfermline, UK). Digital color fundus photography was carried out with the FF450 + IR fundus camera (Carl Zeiss Meditec, Jena, Germany) or the Optos 200 Tx (PLC, Dunfermline, UK). The images were cropped to a 55‐degree field of view for data presentation.

The ffERG was conducted in accordance with international standard settings by the International Society for Clinical Electrophysiology of Vision (ISCEV) utilizing Dawson, Trick, and Litzkow (DTL) electrodes and Ganzfeld stimulation (McCulloch *et al*, 2015). The Diagnosys Espion Electrophysiology System (Diagnosys LLC, Littleton, MA, USA) was used for the ffERG recording.

### Mouse phenotyping

Wild‐type C57BL/6J mice (#000664) were purchased from the Jackson Laboratory and housed in a pathogen‐free environment on a 12‐h light/dark cycle. All mouse experiments in this study were conducted at Columbia University and complied with the Institutional Animal Care and Use Committee (IACUC) and the Association for Research in Vision and Ophthalmology Statement for the Use of Animals in Ophthalmic and Visual Research (Protocol No. AC‐AABD0556, AC‐AABE6581). Deferoxamine mesylate (Toronto Research Chemicals, D228980) was intraperitoneally injected into mice of both genders (100 mg/kg) three times a week starting at weaning age. AKG was dissolved in drinking water at 10 g/l and titrated to ~ pH 7.3 before being supplemented.

SW‐AF (488 nm excitation; Spectralis HRA; Heidelberg Engineering, Heidelberg, Germany) images were captured with a 55‐degree wide field lens over a course of time to assess the RPE lesions following the manufacturer's protocol. SD‐OCT was acquired using an Envisu UHR2200 (Bioptigen, Durham, NC) with theoretical axial resolution in the tissue of 1.75 μm. A rectangular scan with a 1.8‐mm length and width, 0° angle, 0‐mm horizontal and vertical offsets, 1,000 lines of A‐scans/B‐scans, 100 B‐scans, 10 frames/B‐scan, 80 lines of inactive A‐scans/B‐scan, and one volume was captured. Ten‐frame OCT images were averaged using Bioptigen InVivoVue® prior to further processing by Bioptigen Diver^®^ V. 3.4.4 software.

The ERG recording was performed by using the Colordome system (Diagnosys LLC; Lowell, MA, USA). The mice were dark adapted overnight before the test. The c‐wave was recorded with a series of flashing stimuli (0, 0.6, 1.4, 2.17 log cd·s/m^2^) by referring to a previous report (Kinoshita & Peachey, 2018). A digital band‐pass filter ranging from 0.125 to 100 Hz was used to isolate signals after the waves were recorded. C‐wave amplitude was measured from the negative trough after the b‐wave to the peak of the c‐wave. Upon completing c‐wave recording, two steps of dark‐adapted responses were recorded at stimulus levels of −3 and 0.471 log cd·s/m^2^ to represent scotopic, mesopic and photopic responses. Finally, a 10‐min light adaptation was carried out by exposing the mice to a full‐field 30 cd/m^2^ white background, followed by single‐flash stimuli at 1.48 log cd·s/m^2^. A digital band‐pass filter ranging from 0.3 to 300 Hz was used to isolate signals after the waves were recorded. A‐wave amplitude was measured from the baseline to the trough of the a‐wave; b‐wave amplitude was measured from the trough of the a‐wave to the peak of the b‐wave.

### Stem cell culture and *in vitro* assays

iRPE cells were derived from iPSCs in our inventory established from healthy donors. The biopsy samples were obtained according to Columbia University IRB protocol AAAF1894. Informed written consent was obtained. The experiments were performed by conforming to the principles set out in the WMA Declaration of Helsinki and the Department of Health and Human Services Belmont Report. Karyotyping was performed on the iPSCs to exclude chromosomal abnormality. Potential mycoplasma contamination was ruled out in both iPSCs and iRPE by Mycoplasma PCR detection kit (Applied Biological Materials Inc., G238). The cells were seeded at the density of 1 × 10^5^ onto the growth area of 0.32 cm^2^. Deferoxamine mesylate salt (Millipore Sigma, D9533‐1G) and α‐ketoglutaric acid (Millipore Sigma, K1128) were dissolved in culture media containing 0.1% DMSO. The iRPE cells were treated with DFO at 100 mM. The pH of AKG was titrated to ~ 7 and supplemented at 10 mM. The cells provided with culture media containing 0.1% DMSO were included as untreated controls. The assays were conducted 24 and 48 h post treatment.

*Cell viability* was examined by adding Tetrazolium (Invitrogen M6494) solution (0.5 mg/ml) to each well following the MTT assay protocol. The cells subject to tetrazolium were incubated for 3 h at 37°C. The final formazan product was dissolved by isopropanol for subsequent absorbance reading at 595 nm with the iMark microplate reader (BIO‐RAD, #1683315). In parallel, a colorimetric LDH assay (Abcam, ab65393) was performed to determine cytotoxicity associated with DFO. The supernatant (10 μl) of each well from different groups was collected and immediately incubated with LDH Reaction Mix for 40 min. The absorbance was checked at 450 nm. Triplicate readings were set for each group.

*The level of ROS* was measured by the Dihydroethidium (DHE) Assay Kit (Abcam, ab236206). Specifically, the cells were incubated with the DHE assay reagent for 1.5 h at 37°C in a light‐protective environment. Fluorescent signals were checked at the excitation wavelength of 495 nm/emission wavelength of 585 nm. Technical triplicates were set per group for each sample.

*Intracellular ferrous iron* was tested by using the colorimetric Iron Assay Kit (Abcam, ab83366). The cells were thoroughly lysed with the assay buffer and centrifuged at 16,000 *g* for 10 min at 4°C. The supernatant was collected for reacting with the iron probe at 37°C for 1.5 h. Duplicate reading for each group was immediately determined at 595 nm with the iMark microplate reader upon terminating the reaction.

*Metabolic profiles* of the RPE cells were characterized by using the Agilent Seahorse XF Cell Mito Stress Test Kit (Agilent, 103015‐100). The cells were plated onto an Agilent Seahorse XF Cell Culture Microplate (Agilent, 100777‐004) before the measurement. The cells were carefully rinsed and incubated with the designated DMEM media for 40 min. The glucose metabolism assay media was supplemented with 12 mM glucose, 2 mM pyruvate, 2 mM glutamine, and 10 mM HEPES, pH 7.4. DFO and AKG supplements, dissolved in serum‐ and phenol‐free DMEM, were maintained in respective groups during the measurement to conduct a real‐time observation. Acidity due to AKG was carefully neutralized before running the protocol as recommended by the manufacturer. Mitochondrial functions were measured by an XFe24 device with the following mitochondrial modulators added sequentially: 2.5 μM oligomycin, 1 μM carbonyl cyanide p‐trifluoromethoxyphenylhydrazone (FCCP), 0.5 μM antimycin A, and 0.5 μM rotenone. Basal respiration was calculated by subtracting the measurement of oxygen consumption rate (OCR) after the injection of antimycin A and rotenone from that before the injection of oligomycin. Maximal respiration was calculated by subtracting the OCR measurement after antimycin A/rotenone injection from the maximum measurement after FCCP injection. Spare respiratory capacity was calculated by subtracting basal respiration from maximal respiration. ATP production was calculated by subtracting the OCR measurement in the presence of oligomycin from that before any chemical effector modulation. Glycolysis was determined based on the extracellular acidification rate (ECAR) that was automatically generated by the Agilent Seahorse XFe24 Glycolytic Rate Assay Reporter Generator (3.21). A single negative machine readout results in exclusion of the well due to lack of physiological significance.

*Human vascular endothelial growth factor A* (VEGFA) was determined by an enzyme‐linked immunosorbent assay kit (Invitrogen, BMS277‐2) following the manufacturer's protocol. The supernatant collected from the iRPE cells with or without DFO treatment for 24 and 48 h was tested. Technical duplicates were set for each sample per group. Absorbance reading of each microwell was performed at 450 nm with the iMark microplate reader.

### Real‐time qPCR

Total RNA was extracted from iRPE cells using an RNeasy mini kit (QIAGEN, #74104) and was reversely transcribed using SuperScript III First‐Strand Synthesis SuperMix (ThermoFisher, 18080‐400). The reactions were run as previously described (Wang *et al*, 2021). Technical triplicates were set for each biological individual. Transcript levels of each target gene were determined by SYBR Green‐based qPCR (BIO‐RAD, 1725271) and were standardized to *ACTB*. The expression of each transcript was normalized to their pairwise controls. The primer sequences for each target gene are provided in Table 2.

**Table 2:** Oligo primers for real‐time qPCR.

Genes	Sense (5′‐3′)	Anti‐sense (5′‐3′)
*ACTB*	CACCATTGGCAATGAGCGGTTC	AGGTCTTTGCGGATGTCCACGT
*ACSL4*	CAGAAAACTTGGGCATTCCTCC	GCTGGACTGGTCAGAGAGTGT
*ADM*	CCTTCCTAGGCGCTGACACC	ACTGCTGTCTTCGGGGCTT
*BIRC5*	TCTTCTGCTTCAAGGAGCTG	ATGTTCCTCTCTCGTGATCC
*BNIP3*	AAAAACAGCTCACAGTCTGAGG	GCTTCGGGTGTTTAAAGAGGAA
*BNIP3L*	GCTTCGGGTGTTTAAAGAGGAA	TTCTTCATGGCTCCACTTTTCC
*CDKN1A*	GTCACTGTCTTGTACCCTTGTG	GATTAGGGCTTCCTCTTGGAGA
*CHAC1*	GATGCCTGGCCGTGTGGTGA	GTTCTGTGGGGTGGCCACAT
*EPAS1*	GTGCTCCCACGGCCTGTA	TTGTCACACCTATGGCATATCACA
*EPO*	GAGCCCAGAAGGAAGCCATCT	TCTGTCCCCTGTCCTGCAGG
*HIF1A*	CCACAGGACAGTACAGGATG	TCAAGTCGTGCTGAATAATACC
*HK1*	CTGCTGGTGAAAATCCGTAGTGG	GTCCAAGAAGTCAGAGATGCAGG
*HK2*	CCAGTTCATTCACATCATCAG	CTTACACGAGGTCACATAGC
*IGFBP3*	CTGCCGTAGAGAAATGGAAGAC	CCATACTTATCCACACACCAGC
*MYC*	CTTCTCTCCGTCCTCGGATTCT	GAAGGTGATCCAGACTCTGACCTT
*NFE2L2*	CTACTCCCAGGTTGCCCACATT	GAAGTTTCAGGTGACTGAGCCT
*PKM1*	CAGCCAAAGGGGACTATCCT	GAGGCTCGCACAAGTTCTTC
*PKM2*	CTATCCTCTGGAGGCTGTGC	GTGGGGTCGCTGGTAATG
*PTGS2*	GCCATGGGGTGGACTTAAATCA	CAGACCAGGCACCAGACCAA
*SLC2A1*	CGGGCCAAGAGTGTGCTAAA	TGACGATACCGGAGCCAATG
*SLC2A2*	ATGTCAGTGGGACTTGTGCTGC	AACTCAGCCACCATGAACCAGG
*TF*	TGGGCCTGCTCTACAATAAGAT	GCCGTAGTATCCCTCTTTGTTG
*TFRC*	CAAAGACAGCGCTCAAAACTC	TTTTCCCTGCTCTGACAATCAC
*VEGFA*	CCTCCGAAACCATGAACTTT	CCACTTCGTGATGATTCTGC

### Immunoblotting analysis

Cultured iRPE cells were separately prepared for different purposes: First, iRPE cells were homogenized and lysed by 1× Laemmli buffer (BIORAD, #1610747) immediately upon terminating the DFO treatment due to rapid degradation of HIF1α (1:1,000, Novus Biologicals, NB100‐105) and HIF2α (1:2,000, Cell Signaling, #7096; 1:1,000, Novus Biologicals, NB110‐122) in the ambient environment; Second, to extract proteins from different fractions, iRPE cells were resuspended in pre‐chilled 1× phosphate buffered saline (PBS). Part of the resuspended cells were lysed with 1× RIPA buffer as whole cell lysate for detecting PARP (1:1,000, Cell Signaling, #9542). The remaining cell suspension was subject to centrifugation at ~ 750 *g* for 5 min. The pellet was resuspended in 1× Laemmli buffer to extract nuclear content. Immunoblotting with tissue extract was performed by enucleating eyeballs from the mice subject to DFO with and without AKG treatment for 10 months. The RPE was carefully dissected and isolated from the posterior chamber and swiftly lysed in 1× Laemmli buffer to minimize the degradation of HIFα. The protein lysate was resuspended and the supernatant was collected and subject to SDS‐polyacrylamide gel electrophoresis using 4–15% BIO‐RAD TGX pre‐cast gels (#4561083). The proteins were transferred to nitrocellulose membranes for western blotting analysis. Whole‐cell proteins were normalized to β‐actin (1:5,000, Cell Signaling, #3700). Nuclear content was normalized to Histone H3 (1:1,000, Cell Signaling, #9715). Immunoblotting signals were visualized by an iBright FL 1500 Imaging System (ThermoFisher Scientific).

### Microscopic examinations

iRPE cells were seeded on coverslips prior to microscopic examination. The gross morphology was determined using the light microscope Nikon ECLIPSE Ts2R. Fluorescence microscopy was carried out on 2% PFA‐fixed iRPE cells, using ZO‐1 (1:500, Cell signaling, #5406 S). Roche *In Situ* Cell Death Detection Kit, Fluorescein (Millipore Sigma, 11684795910) was used to detect cell death based on the provided protocol. The iRPE was subject to nuclear staining by Hoechst.

Retina and RPE were carefully dissected from the mouse eyes subject to DFO treatment. Untreated mice of similar ages were euthanized as the controls. The eyes were fixed by 4% PFA prior to immunofluorescence staining. The retina was stained with Arrestin 3 (1:400, Millipore Sigma, AB15282) for cone photoreceptors. The RPE was stained with phalloidin (1:1,000, Abcam, ab176756), ZO1 (1:150, Invitrogen, #61‐7300) and IBA1 (1:500, Fujifilm Wako, 019‐19741). Cell death was detected by the TUNEL assay kit (ThermoFisher Scientific, C10617) following the manufacturer's protocol with slight modifications, including extending the TdT reaction to overnight at 37°C. Both retina and RPE were counterstained with Hoechst.

All the immunofluorescence images were acquired with the Nikon A1 HD25 confocal microscope. The pictures were processed by ImageJ and GIMP 2.10.28.

### Statistical analysis

A two‐tailed Student's *t*‐test was performed to calculate the statistical significance between two groups: a ratio paired comparison was considered for using iPSCs as the template. An unpaired comparison was performed for determining statistical significance with mouse samples as the template. One‐way ANOVA with a post‐hoc Tukey's multiple comparison test was performed for calculating the differences among multiple groups. Statistical significance was calculated by GraphPad Prism 9. At least three independent biological replicates were included for statistical calculations unless otherwise stated. Results are shown as mean ± S.E.M., *P* < 0.05 was considered statistically significant.

**The paper explained**

**Problem**

Iron chelation is indispensable for blood transfusion‐dependent patients. Deferoxamine (DFO), an iron chelator is extensively used in the clinic. Retinal degeneration associated with DFO is rare but significantly threatens the patients' visual function. The pathohistological basis of DFO‐related retinal degeneration is not clearly known.

**Results**

In this study, we provided clinical evidence to support retina pigment epithelium (RPE) as a primary target for DFO's toxic effects in the retina. Our experimental characterization further delineated prominent damage to the RPE cells due to the presence of DFO both *in vivo* and *in vitro*. Intriguingly, we identified upregulation of HIF2α and defective mitochondrial function that underlie cell death and atrophy in the RPE. Supplementation of α‐ketoglutarate, an intermediary metabolite of the Krebs cycle, downregulates HIF2α and preserves mitochondrial capacity, alleviating RPE damage in DFO‐related retinopathy.

**Impact**

Our study displays a new line of evidence about the impact of iron depletion on RPE atrophy and retinal degeneration as a clinical consequence. Close examinations of visual function, especially at the early stage are critical to the patients subject to chelation therapy. Inhibiting HIF2α and protecting mitochondrial function may effectively delay the progression of retinal degeneration in the clinic.

## Author contributions

**Yang Kong:** Conceptualization; data curation; formal analysis; validation; investigation; visualization; methodology; writing – original draft; writing – review and editing. **Pei‐Kang Liu:** Data curation; formal analysis; validation; investigation; visualization; methodology; writing – original draft. **Yao Li:** Resources; investigation; methodology. **Nicholas D Nolan:** Formal analysis; investigation; visualization; writing – review and editing. **Peter M J Quinn:** Data curation; investigation; visualization; methodology. **Chun‐Wei Hsu:** Validation; investigation; visualization; methodology. **Laura A Jenny:** Formal analysis; visualization; project administration; writing – review and editing. **Jin Zhao:** Investigation; methodology; project administration; writing – review and editing. **Xuan Cui:** Conceptualization; investigation; methodology. **Ya‐Ju Chang:** Data curation; visualization; methodology. **Katherine J Wert:** Conceptualization; writing – review and editing. **Janet R Sparrow:** Resources; formal analysis; supervision; funding acquisition; methodology; writing – review and editing. **Nan‐Kai Wang:** Data curation; formal analysis; funding acquisition; investigation; visualization; writing – review and editing. **Stephen H Tsang:** Conceptualization; resources; supervision; funding acquisition; methodology; project administration; writing – review and editing.

## Disclosure and competing interest statement

Stephen H Tsang receives research support from Abeona Therapeutics, Inc. and Emendo. He is also the founder of Rejuvitas and is on the advisory board for Nanoscope Therapeutics. Peter M J Quinn receives research support from Rejuvitas, Inc. The other authors have declared that no conflict of interest exists.

## Supporting information



Appendix

Expanded View Figures PDF

Source Data for Expanded View

PDF+

Source Data for Figure 3

Source Data for Figure 4

Source Data for Figure 7

## Data Availability

This study deposits no data in external repositories.
