# Supplementary material for: HIF2α activation and mitochondrial deficit due to iron chelation cause retinal atrophy
Source: EMBO Mol Med. 2023 Jan 16;15(2):e16525. doi: 10.15252/emmm.202216525 (PMC9906391; doi:10.15252/emmm.202216525)
Supplement: Supplementary file 1 — Appendix [file EMMM-15-e16525-s003.pdf]

Table of Contents

Appendixes	About
Figure S1	Supplementary IHC and ELISA data
Table S1	Blood tests for the ferritin

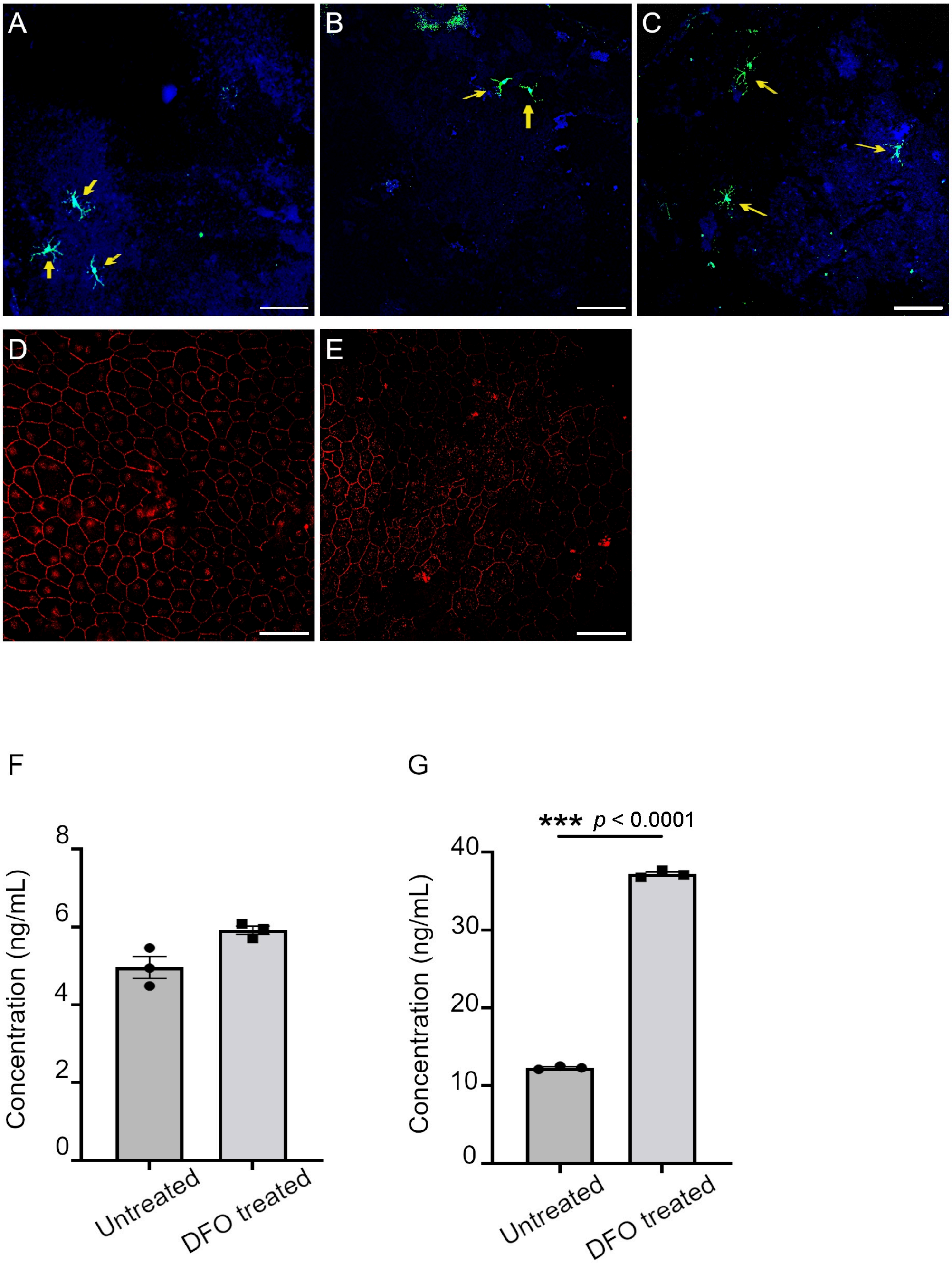
A
B
C
D
E
F
G
Concentration (ng/mL)
8
6
4
2
0
Untreated
DFO treated
*** p < 0.0001
40
30
20
10
0
Concentration (ng/mL)
Untreated
DFO treated

Appendix Figure S1

(A) Representative images of the immunohistochemistry (IHC) against IBA1 to detect the presence of microglia in the mouse RPE collected from the one-year-old mice subject to DFO treatment for 10 months. Scale bar: 100 µm.

(B, C) The RPE of the age-matched C57BL/6J mice was used as the control. Scale bar: 100 µm.

(D) Representative images of the IHC against ZO1 to characterize RPE cell morphology in the mice subject to DFO treatment for 10 months. Scale bar: 50 µm.

(E) Age-matched RPE from the C57BL/6J mice was used as the control. Scale bar: 50 µm.

(F) Measurement of secreted VEGFA by enzyme linked immunosorbent assay in the cultured iRPE cells subject to DFO treatment for 24 hours.

(G) Measurement of secreted VEGFA by enzyme linked immunosorbent assay in the cultured iRPE cells subject to DFO treatment for 48 hours.

Data information: (F, G) Paired student's *t*-test was used for analyzing statistical significance. The results are presented as mean ± S.E.M. n = 4 iRPE lines for each group. *** p <0.0001.

Case I

Date	Blood test	Test results (ng/mL)
4-25-04	Ferritin	340
7-20-04	Ferritin	358
11-9-04	Ferritin	309
2-15-05	Ferritin	386
5-10-05	Ferritin	336
5-24-05	Ferritin	332
8-30-05	Ferritin	492
11-30-05	Ferritin	524
2-21-06	Ferritin	437.5
4-4-06	Ferritin	603.8
4-13-06	Ferritin	638.5
5-2-06	Ferritin	1017
5-23-06	Ferritin	1086.9
6-13-06	Ferritin	835.1
8-8-06	Ferritin	1287.1
9-12-06	Ferritin	1283.7
10-10-06	Ferritin	1339
11-7-06	Ferritin	1383.6
12-5-06	Ferritin	1367.8
1-4-07	Ferritin	1404.2
1-30-07	Ferritin	1427.8
2-27-07	Ferritin	1416
3-27-07	Ferritin	1403.5
5-8-07	Ferritin	1410
6-12-07	Ferritin	1383
7-17-07	Ferritin	1429.5
8-1-07	Ferritin	1801
8-28-07	Ferritin	1357.5
9-11-07	Ferritin	1228.4
10-9-07	Ferritin	935.3
11-6-07	Ferritin	956.1
12-4-07	Ferritin	1060.9
1-2-08	Ferritin	964.5
1-15-08	Ferritin	780
2-14-08	Ferritin	562.2
4-8-08	Ferritin	403.5
6-17-08	Ferritin	331.9
8-12-08	Ferritin	432.1
9-16-08	Ferritin	360
10-7-08	Ferritin	362.4
11-4-08	Ferritin	476.7
12-2-08	Ferritin	440.6
1-13-09	Ferritin	587.8
2-10-09	Ferritin	540.2
3-10-09	Ferritin	583.9
5-5-09	Ferritin	454.8
5-21-09	Ferritin	449.2
8-11-09	Ferritin	442.9
8-25-09	Ferritin	446.4
11-17-09	Ferritin	413
2-9-10	Ferritin	312.1
5-4-10	Ferritin	261.8
8-3-10	Ferritin	479
9-7-10	Ferritin	358.5
11-2-10	Ferritin	277.9
2-10-11	Ferritin	462.2
**3-3-11**	**Ferritin**	**498.8**
5-18-11	Ferritin	622.8
6-14-11	Ferritin	737.1
6-18-11	Ferritin	861.8
6-20-11	Ferritin	1014
6-21-11	Ferritin	880.1
6-22-11	Ferritin	839.8
6-23-11	Ferritin	916.2
6-28-11	Ferritin	822.7
7-12-11	Ferritin	667.6
8-30-11	Ferritin	668.6
10-11-11	Ferritin	725.2
11-8-11	Ferritin	819.1
12-31-11	Ferritin	825
1-10-12	Ferritin	830.7
2-29-12	Ferritin	663.9
4-24-12	Ferritin	834.6
6-13-12	Ferritin	973.1
7-30-12	Ferritin	1030.3
9-3-12	Ferritin	982.9
9-19-12	Ferritin	1145.2

Case II

Date	Blood test	Test results (ng/mL)
10-8-02	Ferritin	2107
4-22-03	Ferritin	2354
10-7-03	Ferritin	2286
4-6-04	Ferritin	1193
10-12-04	Ferritin	1414
4-8-05	Ferritin	1409
10-28-05	Ferritin	1136
6-2-06	Ferritin	1570
11-10-06	Ferritin	977.2
2-2-07	Ferritin	1630
2-27-07	Ferritin	1567
3-20-07	Ferritin	1321
4-13-07	Ferritin	1266
5-4-07	Ferritin	2738
6-1-07	Ferritin	3478
6-22-07	Ferritin	2087
7-13-07	Ferritin	2575
8-3-07	Ferritin	2293
10-26-07	Ferritin	1554
2-15-08	Ferritin	2882
7-7-08	Ferritin	1605
10-17-08	Ferritin	1412
3-20-09	Ferritin	2256
12-25-09	Ferritin	1517
1-21-11	Ferritin	1346
5-27-11	Ferritin	918.2
8-19-11	Ferritin	1078
**12-23-11**	**Ferritin**	**953.9**
4-27-12	Ferritin	554
8-24-12	Ferritin	1343
12-7-12	Ferritin	3910
5-10-13	Ferritin	3381
10-4-13	Ferritin	2974
3-28-14	Ferritin	5356 (R)
8-8-14	Ferritin	2563
1-16-15	Ferritin	2150
5-19-15	Ferritin	2095
9-4-15	Ferritin	1971
1-29-16	Ferritin	2707
7-15-16	Ferritin	2686
1-24-17	Ferritin	6030
6-16-17	Ferritin	5024
10-20-17	Ferritin	4097
7-13-18	Ferritin	3304
2-1-19	Ferritin	3418
8-30-19	Ferritin	1453
10-4-19	Ferritin	1555
4-10-20	Ferritin	2031
11-23-20	Ferritin	1845
8-6-21	Ferritin	1523
12-10-21	Ferritin	1805
5-27-22	Ferritin	1118

Case III

Date	Blood test	Test results (ng/mL)
**2012-08-10**	**Ferritin**	**439**
8-2-18	Ferritin	1650
8-24-18	Ferritin	1358
9-7-18	Ferritin	1627
9-21-18	Ferritin	1102
10-5-18	Ferritin	1650
10-19-18	Ferritin	1258
11-21-18	Ferritin	1340
12-7-18	Ferritin	1335
12-21-18	Ferritin	1459
1-18-19	Ferritin	1268
1-31-19	Ferritin	1270
2-15-19	Ferritin	1558
2-28-19	Ferritin	1650
3-15-19	Ferritin	1540
3-29-19	Ferritin	1571
4-12-19	Ferritin	1744
4-26-19	Ferritin	1650
5-17-19	Ferritin	1452.6
6-14-19	Ferritin	1650
6-28-19	Ferritin	1648.8
7-12-19	Ferritin	2070
7-26-19	Ferritin	1689
8-9-19	Ferritin	2042
8-28-19	Ferritin	1848
9-13-19	Ferritin	1655.3
9-27-19	Ferritin	1633
10-11-19	Ferritin	1806
10-25-19	Ferritin	1573.4
11-8-19	Ferritin	1547
11-22-19	Ferritin	1821
12-12-19	Ferritin	1775
12-27-19	Ferritin	1650
2-13-20	Ferritin	2500.4
3-6-20	Ferritin	1376
3-20-20	Ferritin	1432
4-3-20	Ferritin	1554
5-1-20	Ferritin	1650
5-15-20	Ferritin	1795
5-29-20	Ferritin	1774
6-12-20	Ferritin	1951.2
6-26-20	Ferritin	1909.2
7-10-20	Ferritin	1983.9
7-24-20	Ferritin	1935

Case IV

Date	Blood test	Test results (ng/mL)
3-22-17	Ferritin	305.3
5-9-17	Ferritin	323
6-27-17	Ferritin	286
8-10-17	Ferritin	304
11-6-17	Ferritin	261
12-19-17	Ferritin	299
1-19-18	Ferritin	324
2-20-18	Ferritin	332
5-30-18	Ferritin	306
8-7-18	Ferritin	279
11-13-18	Ferritin	331
**12-11-18**	**Ferritin**	**342**
2-19-19	Ferritin	328
5-17-19	Ferritin	250
8-28-19	Ferritin	243
12-17-19	Ferritin	327
1-27-20	Ferritin	328
4-14-20	Ferritin	354
7-9-20	Ferritin	261
9-22-20	Ferritin	365
12-14-20	Ferritin	391
2-28-21	Ferritin	503
4-15-21	Ferritin	509
7-1-21	Ferritin	396
8-23-21	Ferritin	418
11-9-21	Ferritin	439
3-4-22	Ferritin	575
6-9-22	Ferritin	328
9-8-22	Ferritin	481
10-6-22	Ferritin	514

Normal Range (male, ng/mL)
23.9 - 336.2

Appendix Table S1. The patients' blood test results of ferritin over the course of chelation therapy. The most recent test of ferritin before their initial visit to the clinic of ophthalmology was highlighted in bold.
