# Supplementary material for: HIF2α activation and mitochondrial deficit due to iron chelation cause retinal atrophy
Source: EMBO Mol Med. 2023 Jan 16;15(2):e16525. doi: 10.15252/emmm.202216525 (PMC9906391; doi:10.15252/emmm.202216525)
Supplement: Supplementary file 2 — Expanded View Figures PDF [file EMMM-15-e16525-s005.pdf]

Expanded View Figures

Figure EV1. The chelation-dependent thalassemia patients show retinal degeneration and functional improvement by taking AKG.

A–H SD-OCT of the four thalassemia patients with a history of taking DFO showed interruption of the ellipsoid zone (Case I; Gelman *et al*, 2014), decreased photoreceptor nuclear layer, and thinning of the retinal layers (Cases II–IV). Focal thickening and bumps of RPE were also noted in Case I. Granular hyper-reflective deposits within the RPE (yellow arrows) can be seen in all four cases. An intraretinal degenerative cyst (Case IV) and multiple areas of choroidal hyper-transmission were also noted in Cases II–IV.

I The ffERG examination on Case IV with continuous supplementation of AKG for 18 months (2 g/day). Repeated ffERG examination (lower panel) on both eyes showed increased amplitudes in light-adapted single-flash cone and 30 Hz flicker responses compared with the amplitude prior to taking AKG (upper panel). The exact numbers of the peak value are indicated inside the panel boxes. Y-Axis: microvolts; X-Axis: milliseconds.

© 2023 The Authors

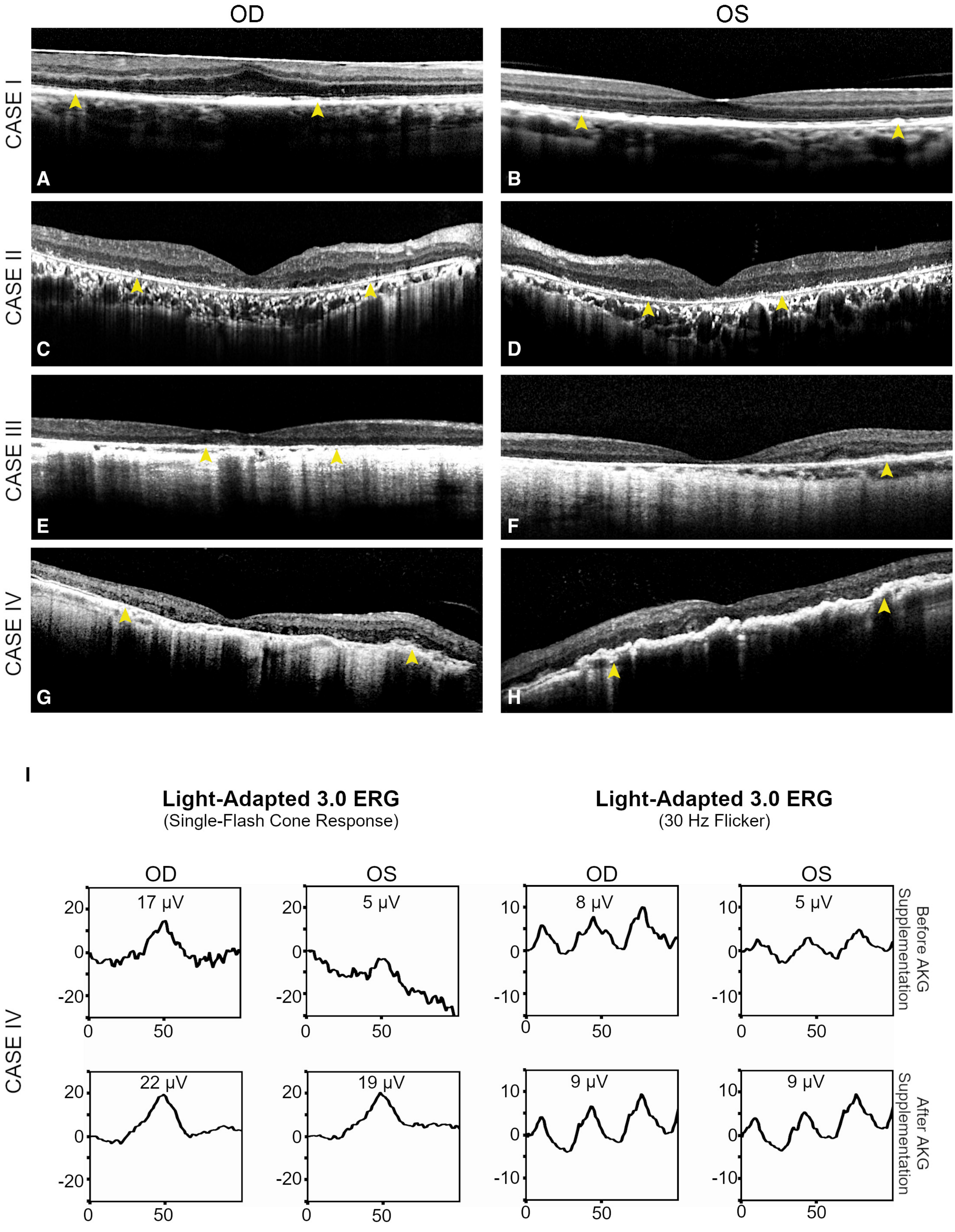

Figure EV1.

 © 2023 The Authors

Figure EV2. The toxic effects of DFO in the outer retina/RPE area.

A, B SD-OCT was performed on DFO-treated mouse eyes to display potential changes to RPE and retina, respectively. Yellow arrows denote hyper-reflective signal and outer retinal lesion in SW-AF and SD-OCT, respectively (A); and hypo-reflective signal and lesions located in the photoreceptor outer segment/RPE microvilli area (B). Yellow lines in the near-infrared images indicate the sections scanned by SD-OCT.

C, D Cell death was examined by TUNEL assay in the retinas collected from the mice with DFO treatment for five months.

E, F Cone photoreceptors were stained by Arrestin 3 in retinal flat mounts from the mice with DFO treatment for five months.

Data information: (C–F) The images were captured at 10× magnification and stitched together. Scale bar: 1 mm.

© 2023 The Authors

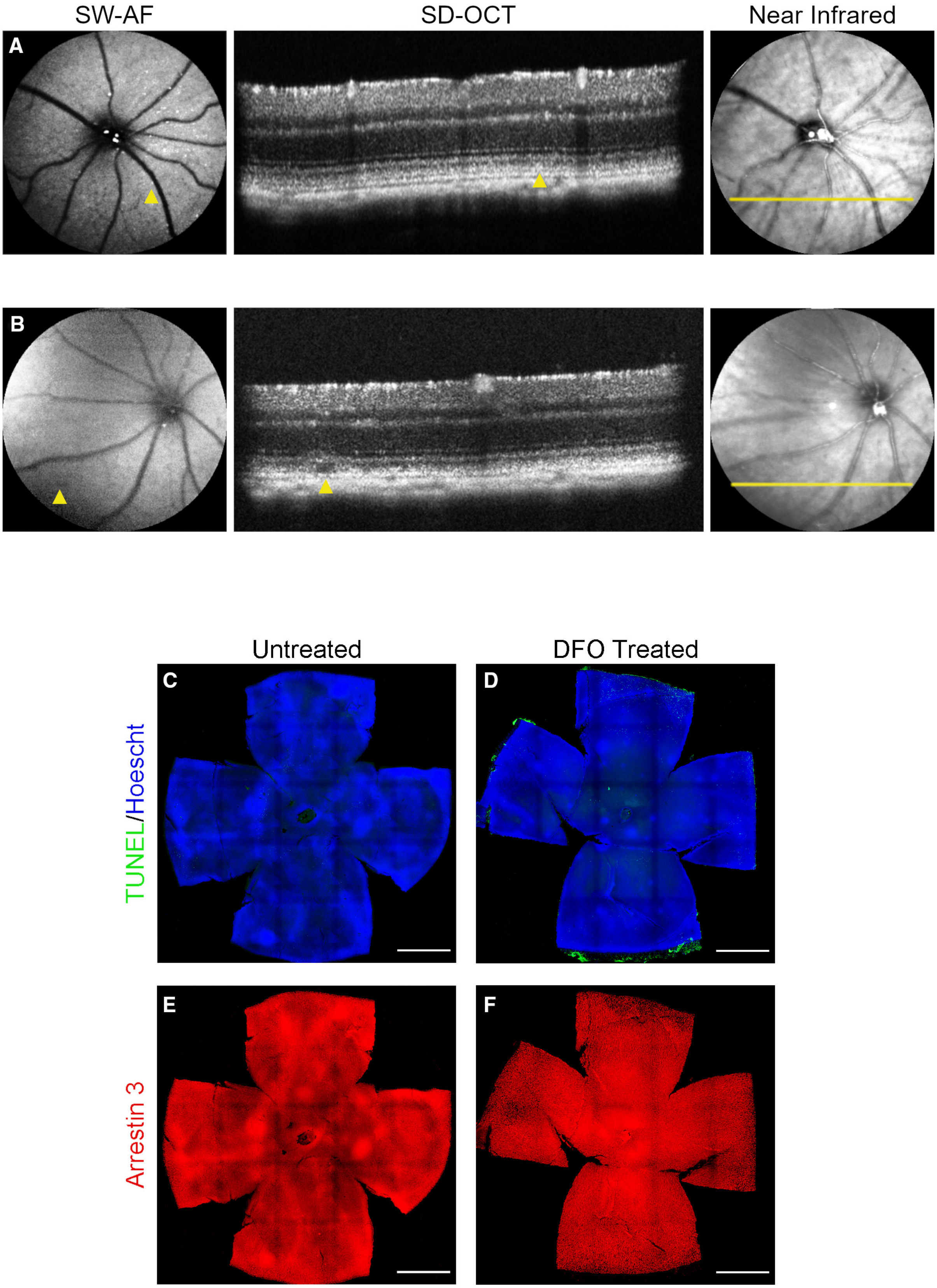

Figure EV2.

 © 2023 The Authors

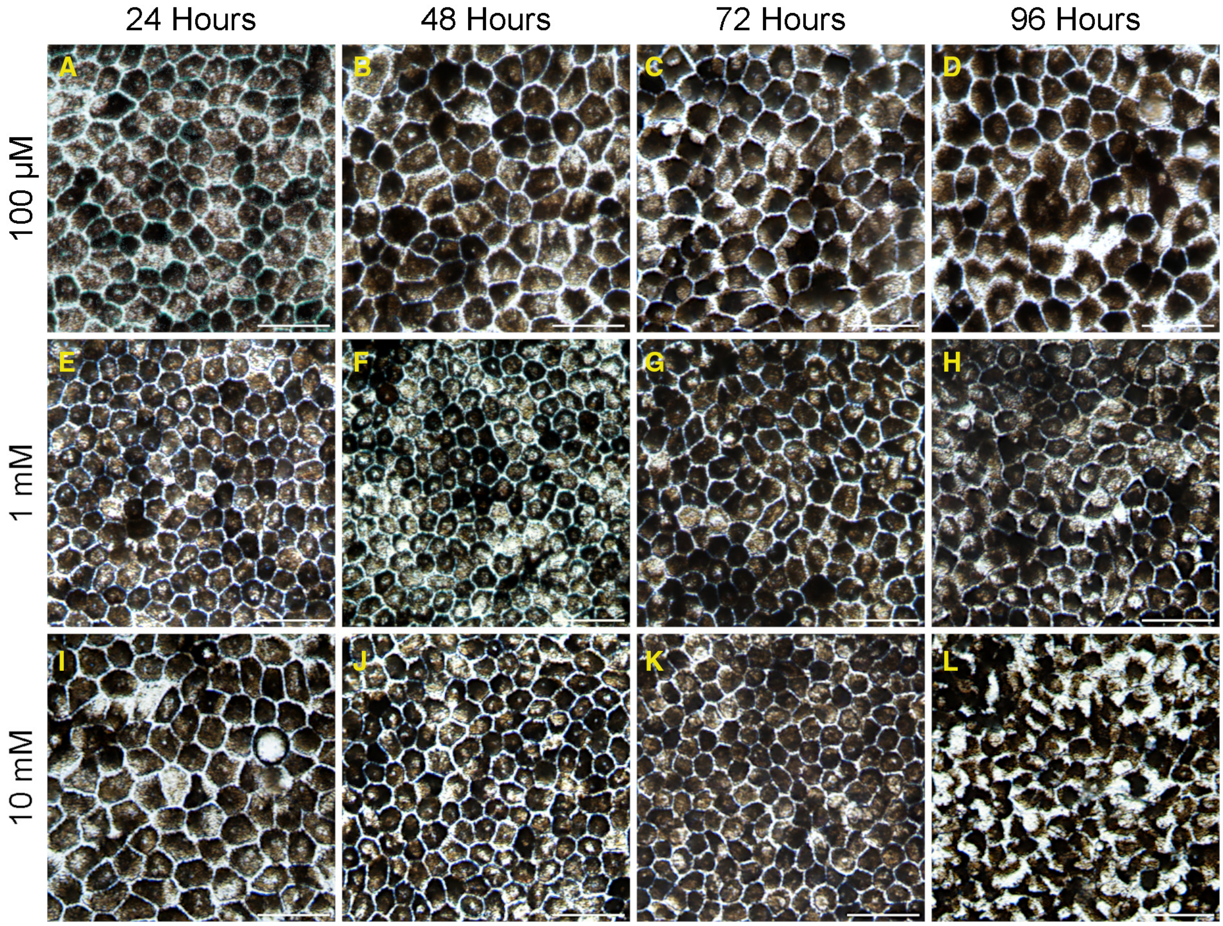

Figure EV3. Examination of the toxicity gradient of DFO in iRPE cells.

A–D The iRPE cells were treated with DFO for different time points at 100 μM.
E–H The iRPE cells were treated with DFO for different time points at 1 mM.
I–L The iRPE cells were treated with DFO for different time points at 10 mM.

Data information: The toxic effect was monitored by light microscopy over a course of time for up to 96-h until the disruption of cell integrity distinctly appeared. Scale bar: 20 μm.
Source data are available online for this figure.

Figure EV4. Measurement of HIFα and its target genes in response to DFO in iRPE cells.

A–E The HIFα-regulated transcripts were determined by qPCR with iRPE cells subject to DFO treatment for 48-h. The cDNA transcript extracted from the untreated iRPE was included as the control. The level of each transcript was normalized to *ACTB*. (A) Measurement of the HIF1α and HIF2α transcripts. The statistics are analyzed by one-way ANOVA with the Tukey test. The results are presented as mean ± S.E.M., $n = 4$ iRPE lines for each group. (B) Measurement of cell-survival-related transcripts regulated by HIFα. (C) Measurement of apoptosis-related transcript regulated by HIFα. (D) Measurement of glycolysis-related transcript regulated by HIFα. (E) Measurement of iron-transport-related transcript regulated by HIFα.

F RPE lysate from the one-year-old mice subject to 10-month DFO treatment with or without concomitant supplementation of AKG was used for immunoblotting against HIF1α. The RPE harvested from the age-matched untreated mice was included as the control. HIF1α (degraded) was predicted to be 40–80 kDa. Actin was used as the loading control.

Data information: (B–E) The statistics are analyzed by ratio paired Student's *t*-test. The results are presented as mean ± S.E.M., $n = 4$ iRPE lines for each group.
$^*P < 0.05$; $^{**}P < 0.01$. Round dots: untreated iRPE; square dots: DFO-treated iRPE.
Source data are available online for this figure.

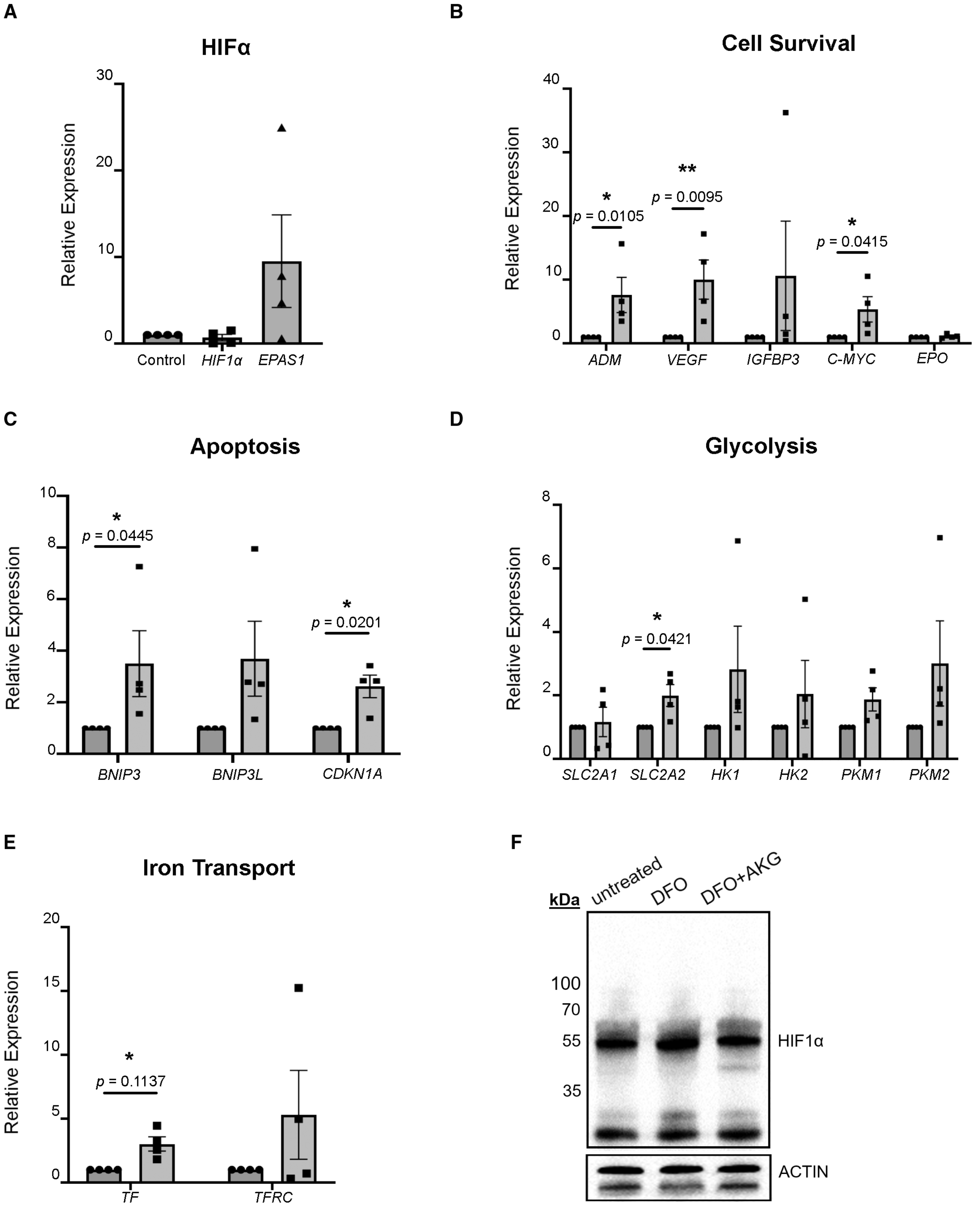

Figure EV4.

 © 2023 The Authors
