# Supplementary figures and images for: HIF2α activation and mitochondrial deficit due to iron chelation cause retinal atrophy
Source: EMBO Mol Med. 2023 Jan 16;15(2):e16525. doi: 10.15252/emmm.202216525 (PMC9906391; doi:10.15252/emmm.202216525)

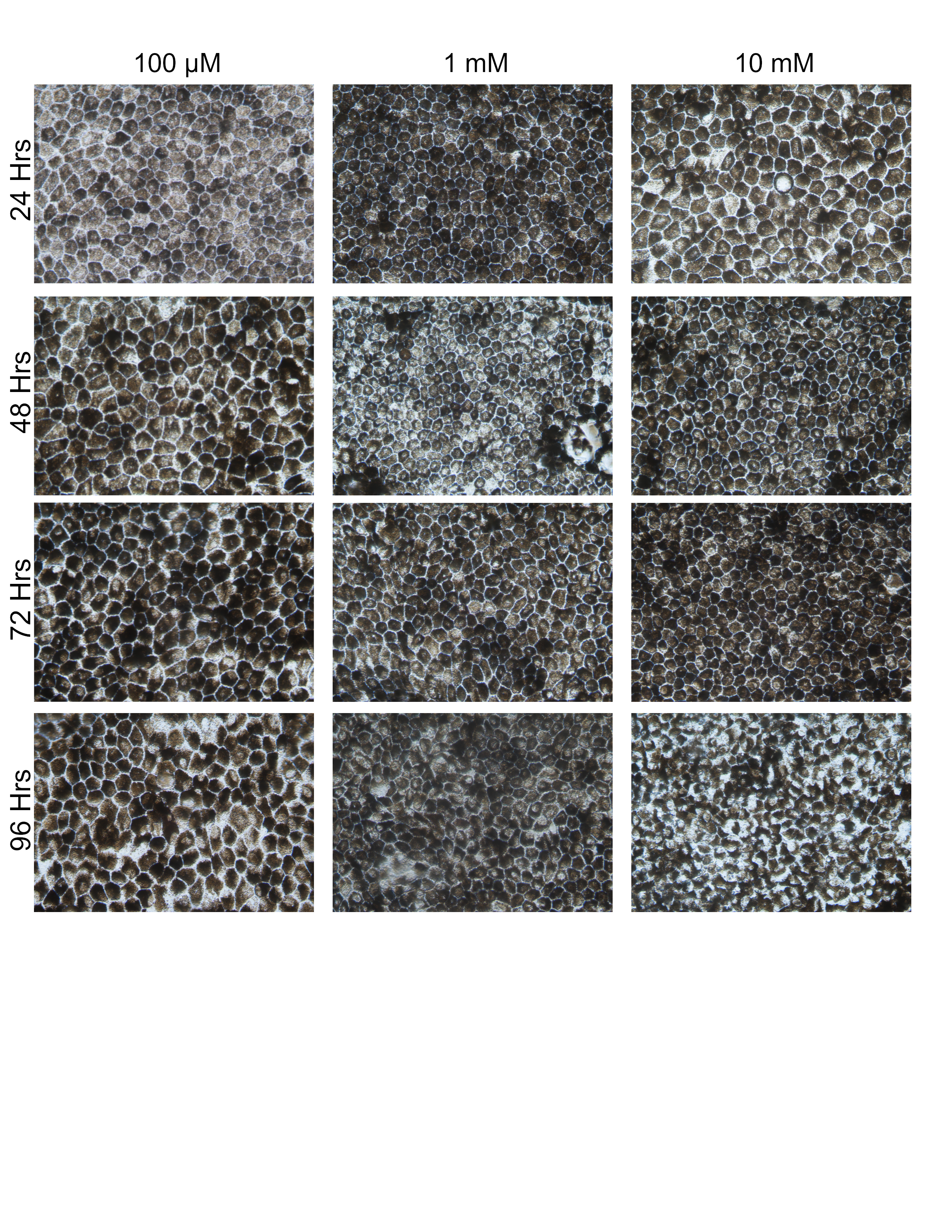

Supplement: Supplementary file 3 — Source Data for Expanded View [file EMMM-15-e16525-s006.zip › EMMM_2431_Source Data Figure EV3.tif]

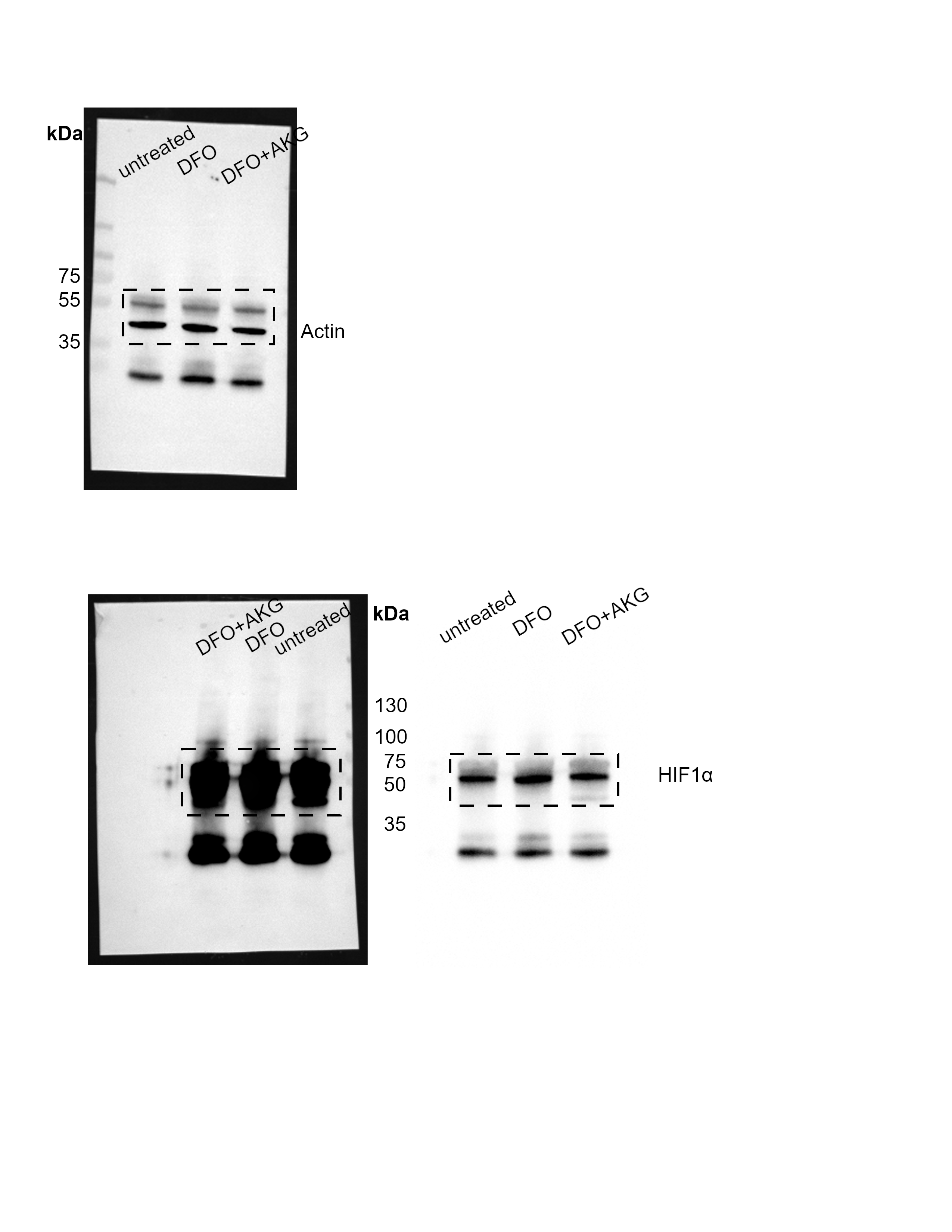

Supplement: Supplementary file 3 — Source Data for Expanded View [file EMMM-15-e16525-s006.zip › EMMM_2431_source data Figure EV4.tif]

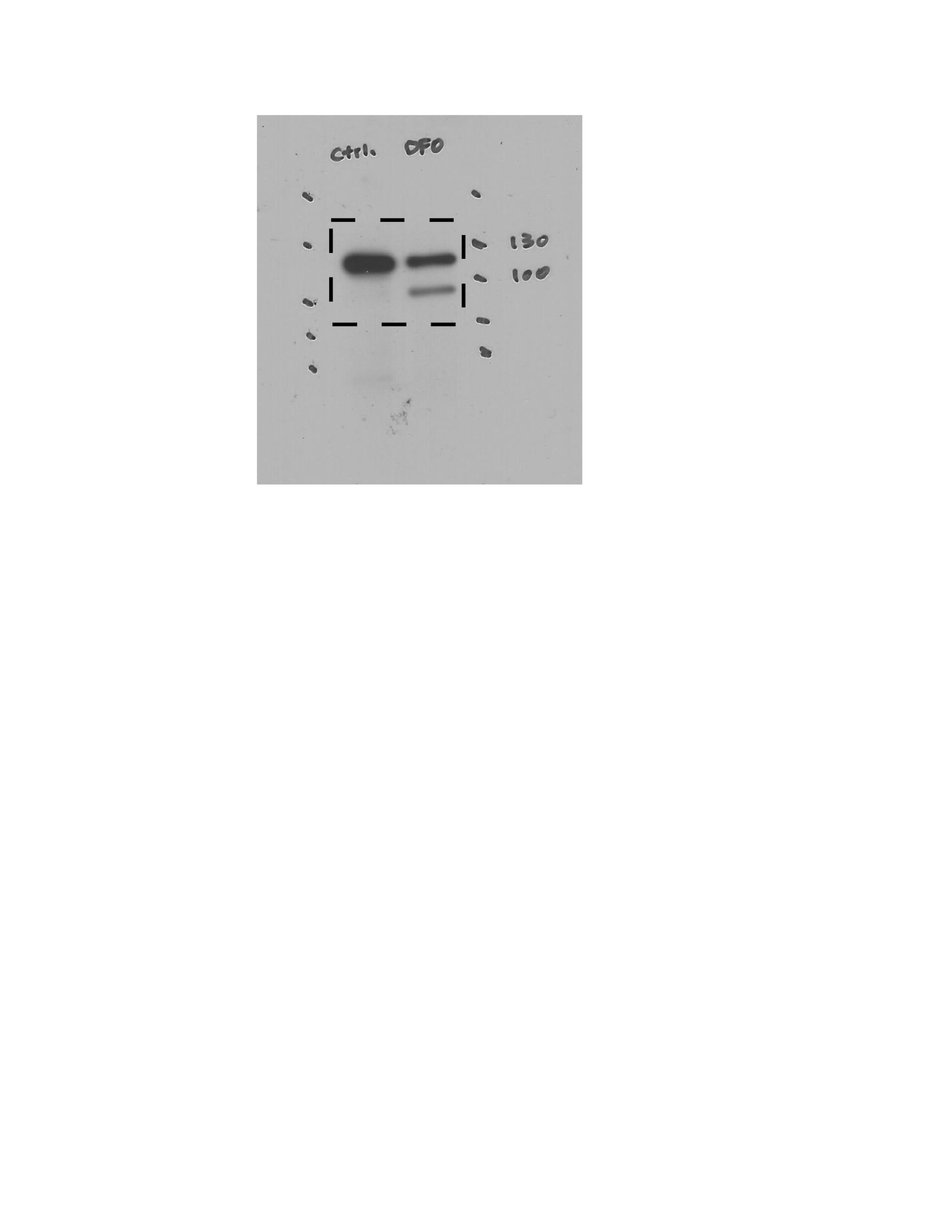

Supplement: Supplementary file 5 — Source Data for Figure 3 [file EMMM-15-e16525-s008.tif]

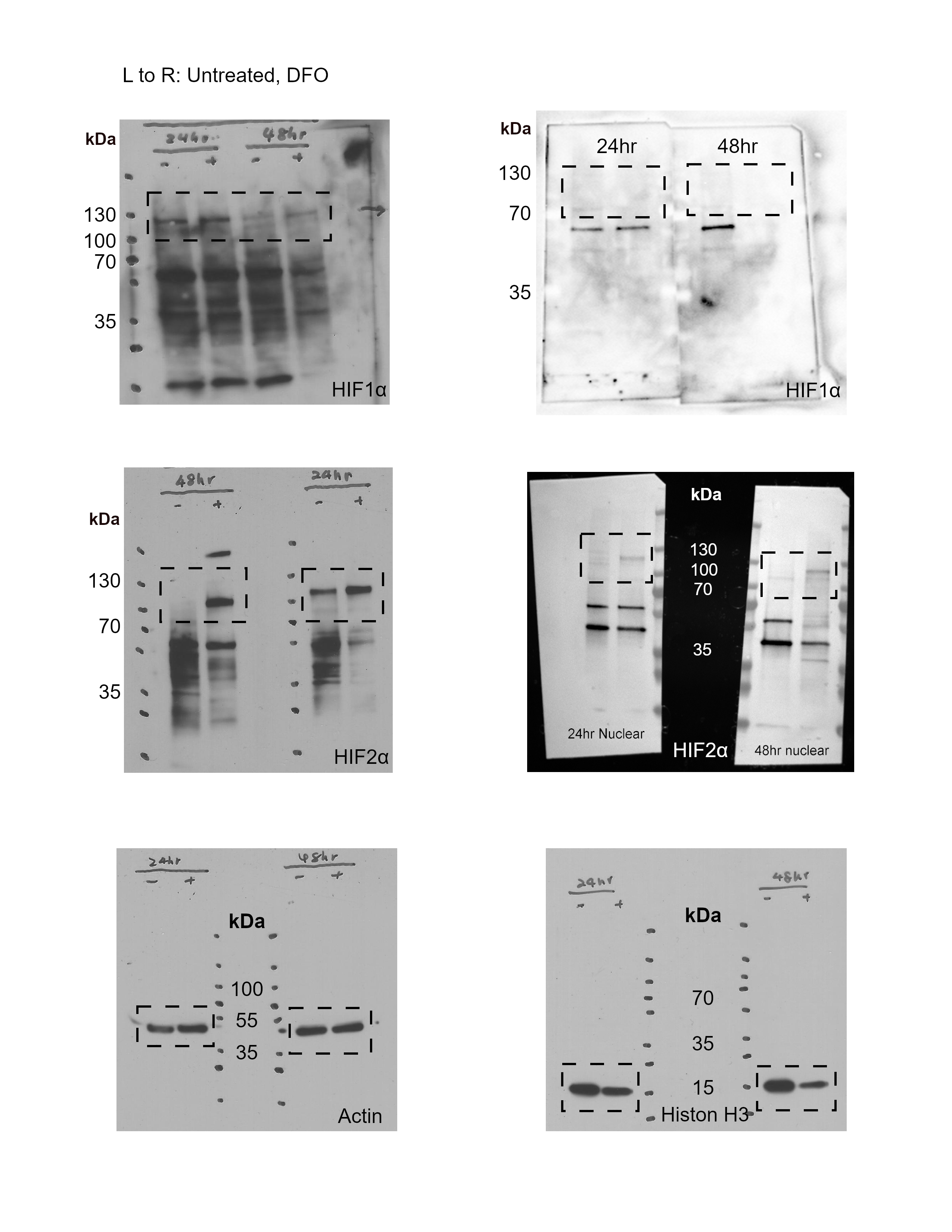

Supplement: Supplementary file 6 — Source Data for Figure 4 [file EMMM-15-e16525-s002.tif]

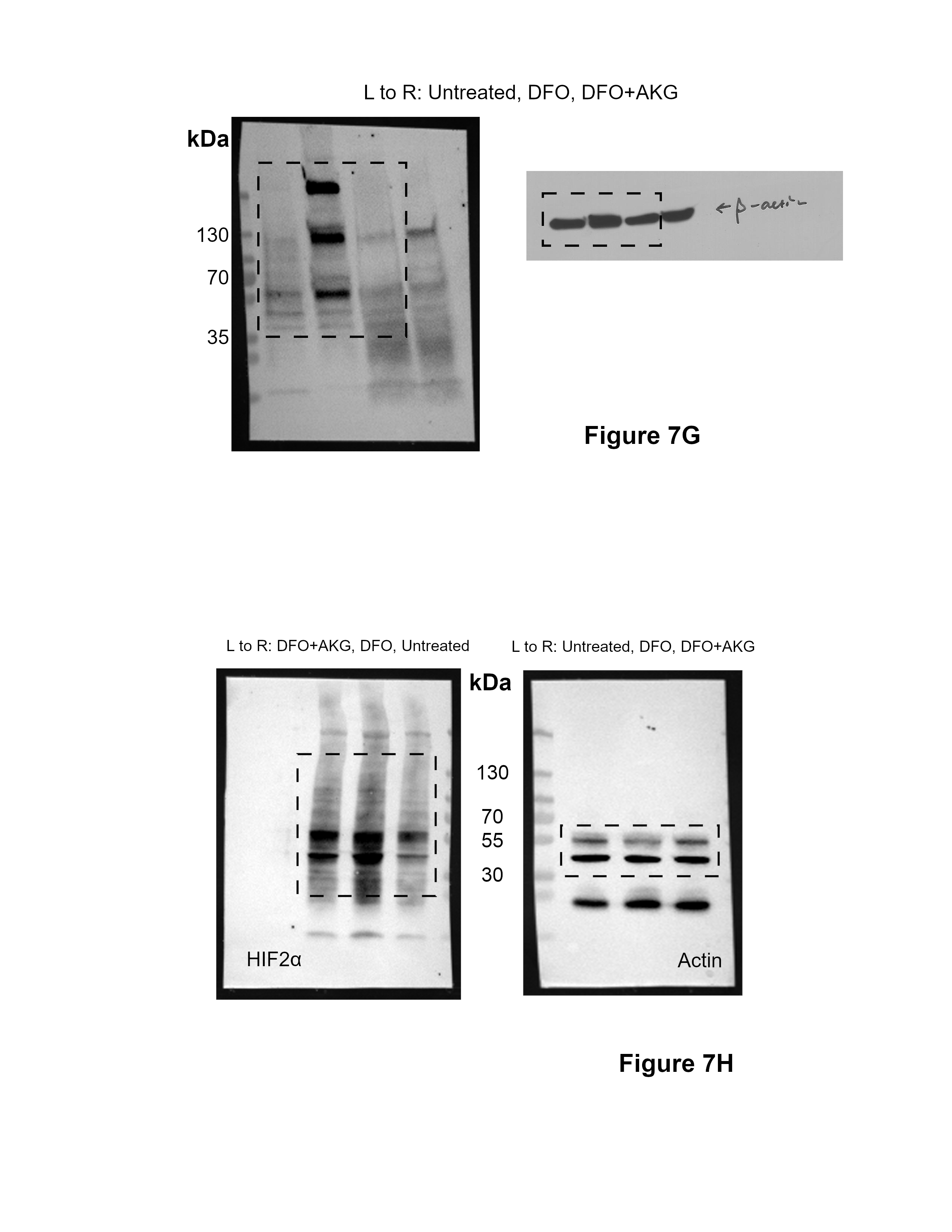

Supplement: Supplementary file 7 — Source Data for Figure 7 [file EMMM-15-e16525-s007.tif]
