# Supplementary material for: HIF2α activation and mitochondrial deficit due to iron chelation cause retinal atrophy
Source: EMBO Mol Med. 2023 Jan 16;15(2):e16525. doi: 10.15252/emmm.202216525 (PMC9906391; doi:10.15252/emmm.202216525)
Supplement: Supplementary file 8 [file EMMM-15-e16525-s001.pdf]

EMBO Molecular Medicine

HIF2α Activation and Mitochondrial Deficit due to Iron Chelation Cause Retinal Atrophy

Yang Kong, Pei-Kang Liu, Yao Li, Nicholas Nolan, Peter Quinn, Chun-Wei Hsu, Laura Jenny, Jin Zhao, Xuan Cui, Ya-Ju Chang, Katherine Wert, Janet Sparrow, Nan-Kai Wang, and Stephen Tsang
DOI: 10.15252/emmm.202216525

Corresponding author: Stephen Tsang (crispr.ips@gmail.com)

Review Timeline:

Submission Date:	1st Jul 22
Editorial Decision:	26th Jul 22
Revision Received:	5th Nov 22
Editorial Decision:	30th Nov 22
Revision Received:	10th Dec 22
Accepted:	13th Dec 22

Editor: Lise Roth

Transaction Report:

(Note: With the exception of the correction of typographical or spelling errors that could be a source of ambiguity, letters and reports are not edited. Depending on transfer agreements, referee reports obtained elsewhere may or may not be included in this compilation. Referee reports are anonymous unless the Referee chooses to sign their reports.)

26th Jul 2022

Dear Dr. Tsang,

Thank you for the submission of your manuscript to EMBO Molecular Medicine. We have now received feedback from the three reviewers who agreed to evaluate your manuscript. As you will see from the reports below, the referees acknowledge the interest of the study and are overall supporting publication of your work pending appropriate revisions.

Addressing the reviewers' concerns in full will be necessary for further considering the manuscript in our journal, and acceptance of the manuscript will entail a second round of review. EMBO Molecular Medicine encourages a single round of revision only and therefore, acceptance or rejection of the manuscript will depend on the completeness of your responses included in the next, final version of the manuscript. For this reason, and to save you from any frustrations in the end, I would strongly advise against returning an incomplete revision.

Revised manuscripts should be submitted within three months of a request for revision; they will otherwise be treated as new submissions, except under exceptional circumstances in which a short extension is obtained from the editor.

When submitting your revised manuscript, please carefully review the instructions that follow below. We perform an initial quality control of all revised manuscripts before re-review; failure to include requested items will delay the evaluation of your revision.

We require:

1) A .docx formatted version of the manuscript text (including legends for main figures, EV figures and tables). Please make sure that the changes are highlighted to be clearly visible.

2) Individual production quality figure files as .eps, .tif, .jpg (one file per figure). For guidance, download the 'Figure Guide PDF' (https://www.embopress.org/page/journal/17574684/authorguide#figureformat).

3) A .docx formatted letter INCLUDING the reviewers' reports and your detailed point-by-point responses to their comments. As part of the EMBO Press transparent editorial process, the point-by-point response is part of the Review Process File (RPF), which will be published alongside your paper.

4) A complete author checklist, which you can download from our author guidelines (https://www.embopress.org/page/journal/17574684/authorguide#submissionofrevisions). Please insert information in the checklist that is also reflected in the manuscript. The completed author checklist will also be part of the RPF.

5) Please note that all corresponding authors are required to supply an ORCID ID for their name upon submission of a revised manuscript.

6) It is mandatory to include a 'Data Availability' section after the Materials and Methods. Before submitting your revision, primary datasets produced in this study need to be deposited in an appropriate public database, and the accession numbers and database listed under 'Data Availability'. Please remember to provide a reviewer password if the datasets are not yet public (see https://www.embopress.org/page/journal/17574684/authorguide#dataavailability).
In case you have no data that requires deposition in a public database, please state so in this section. Note that the Data Availability Section is restricted to new primary data that are part of this study.

7) For data quantification: please specify the name of the statistical test used to generate error bars and P values, the number (n) of independent experiments (specify technical or biological replicates) underlying each data point and the test used to calculate p-values in each figure legend. The figure legends should contain a basic description of n, P and the test applied. Graphs must include a description of the bars and the error bars (s.d., s.e.m.). Please provide exact p values, not a range.

8) We would also encourage you to include the source data for figure panels that show essential data. Numerical data should be provided as individual .xls or .csv files (including a tab describing the data). For blots or microscopy, uncropped images should be submitted (using a zip archive if multiple images need to be supplied for one panel). Additional information on source data and instruction on how to label the files are available at
.

9) Our journal encourages inclusion of *data citations in the reference list* to directly cite datasets that were re-used and obtained from public databases. Data citations in the article text are distinct from normal bibliographical citations and should directly link to the database records from which the data can be accessed. In the main text, data citations are formatted as follows: "Data ref: Smith et al, 2001" or "Data ref: NCBI Sequence Read Archive PRJNA342805, 2017". In the Reference list,

data citations must be labeled with "[DATASET]". A data reference must provide the database name, accession number/identifiers and a resolvable link to the landing page from which the data can be accessed at the end of the reference. Further instructions are available at .

10) We replaced Supplementary Information with Expanded View (EV) Figures and Tables that are collapsible/expandable online. A maximum of 5 EV Figures can be typeset. EV Figures should be cited as 'Figure EV1, Figure EV2" etc... in the text and their respective legends should be included in the main text after the legends of regular figures.
- For the figures that you do NOT wish to display as Expanded View figures, they should be bundled together with their legends in a single PDF file called *Appendix*, which should start with a short Table of Content. Appendix figures should be referred to in the main text as: "Appendix Figure S1, Appendix Figure S2" etc.
- Additional Tables/Datasets should be labeled and referred to as Table EV1, Dataset EV1, etc. Legends have to be provided in a separate tab in case of .xls files. Alternatively, the legend can be supplied as a separate text file (README) and zipped together with the Table/Dataset file.
See detailed instructions here:
.

11) The paper explained: EMBO Molecular Medicine articles are accompanied by a summary of the articles to emphasize the major findings in the paper and their medical implications for the non-specialist reader. Please provide a draft summary of your article highlighting
- the medical issue you are addressing,
- the results obtained and
- their clinical impact.
This may be edited to ensure that readers understand the significance and context of the research. Please refer to any of our published articles for an example.

12) For more information: There is space at the end of each article to list relevant web links for further consultation by our readers. Could you identify some relevant ones and provide such information as well? Some examples are patient associations, relevant databases, OMIM/proteins/genes links, author's websites, etc...

13) Conflict of interest: We updated our journal's competing interests policy in January 2022 and request authors to consider both actual and perceived competing interests. Please review the policy https://www.embopress.org/competing-interests and update your competing interests if necessary.

14) Every published paper now includes a 'Synopsis' to further enhance discoverability. Synopses are displayed on the journal webpage and are freely accessible to all readers. They include a short stand first (maximum of 300 characters, including space) as well as 2-5 one-sentences bullet points that summarizes the paper. Please write the bullet points to summarize the key NEW findings. They should be designed to be complementary to the abstract - i.e. not repeat the same text. We encourage inclusion of key acronyms and quantitative information (maximum of 30 words / bullet point). Please use the passive voice. Please attach these in a separate file or send them by email, we will incorporate them accordingly.
Please also suggest a striking image or visual abstract to illustrate your article as a PNG file 550 px wide x 300-600 px high.

15) As part of the EMBO Publications transparent editorial process initiative (see our Editorial at http://embomolmed.embopress.org/content/2/9/329), EMBO Molecular Medicine will publish online a Review Process File (RPF) to accompany accepted manuscripts.
In the event of acceptance, this file will be published in conjunction with your paper and will include the anonymous referee reports, your point-by-point response and all pertinent correspondence relating to the manuscript. Let us know whether you agree with the publication of the RPF and as here, if you want to remove or not any figures from it prior to publication.
Please note that the Authors checklist will be published at the end of the RPF.

EMBO Molecular Medicine has a "scooping protection" policy, whereby similar findings that are published by others during review or revision are not a criterion for rejection. Should you decide to submit a revised version, I do ask that you get in touch after three months if you have not completed it, to update us on the status.

I look forward to receiving your revised manuscript.

Yours sincerely,

Lise Roth

Lise Roth, PhD
Senior Editor
EMBO Molecular Medicine

***** Reviewer's comments *****

Referee #1 (Remarks for Author):

Deferoxamine treatment, an iron
chelator can have severe adverse effects, including ocular toxicity.
The group here presents interesting data from patients, a mouse model and iPS-derived RPE cells. The paper reports a specific susceptibility of RPE cells to deferoxamine that leads to mitochondrial (HIF2a) dysfunction leading then to RPE atrophy.

I am very enthusiastic about the first parts of the paper that show a clear translational approach in patients and mice. The last part with a-ketoglutarate (AKG) as a direct therapy for RPE damage via HIF2A inhibition is less clear. There have been several papers that citric cycle intermediates can alleviate retinal degeneration in more general setting of IRDs.This point should be at least discussed carefully.

Minor points:

1. There is a hypothesis that hyperreflective spots seen in mouse retinal fundus imaging of damage models could potentially represent reactive immune cells (microglia). It would be worth to have a look on retinal and RPE/choroidal flat mounts stained with markers such as Iba1 to rule out this immune-mediated phenomenon in the mouse damage/treatment studies.
2. Fig. 2K-N. The phalloiding staining of the (damaged) RPE cell network should be supplemented with a second verification marker such as zonula occludens protein 1 (ZO1) (as has been done for in vitro RPE in Fig. 3).
3. I wonder whether the strongly induced expression in the iRPE cells in VEGF transcripts really translates to VEGF-A protein expression, which would also indicate a proangiogenic trigger of deferoxamine.

Referee #2 (Comments on Novelty/Model System for Author):

The major conclusions need to be confirmed in the mouse model. The iRPE data are of unclear validity due to the extremely high concentration of DFO used.

Referee #2 (Remarks for Author):

In this article, the authors address the problem of Deferoxamine (DFO) toxicity on the eye. DFO is an iron chelator in clinical use to remove iron from overloaded patients with thalassemia, especially those dependent on red cell transfusions. Ocular toxicity occurs in less than 10% of patients taking DFO, and it is unclear why they are particularly susceptible and what the mechanism of toxicity is. The manuscript presents clinical data from 4 patients, mouse model experiments and iRPE experiments. The authors come to the surprising conclusion that the toxic effects of DFO are due to iron starvation of the RPE, manifested in particular by mitochondrial respiratory dysfunction and HIF2 alpha induction. The toxic effects could be partially rescued by treatment with alpha keto glutarate, a PHD substrate involved in HIF induction.

Pharmacology - How were the DFO doses used arrived at? The dose for the mice was 100 mg/kg, somewhat higher than the recommended dose for human use, but in the same range. What is the effective drug concentration here? The dose for the iRPE experiments, 100 mM, seems to be a huge excess over the recommended dosing. Toxicity from this high dose may or may not reflect the situation in intact retina in an organism (mouse or human) treated with the recommended DFO dosing. The conclusions regarding the role of mitochondrial respiration and HIF2 alpha induction in DFO toxicity are based on the experiments with iRPE with the extra high DFO concentration. If possible these results should be confirmed in the mouse model. Does the RPE tested in seahorse lose respiratory capacity? Is HIF2 alpha induced in RPE of the mouse treated with DFO. Does AKG rescue in the mouse setting.

A major finding of the paper is that the toxicity of DFO relates to iron starvation. In the write-up on Deferoxamine (Drug information, Up-to-Date), it says "Ocular disturbances ...have been reported following prolonged administration at high doses or in patients with low ferritin levels..." The database goes on to say, "a lower dose may be required if ferritin levels are low. In

general the therapeutic index should be kept at <0.05. Therapeutic index = mean daily deferoxamine dose (mg/kg)/ferritin (mcg/L)." The implication of this recommendation is that DFO toxicity may occur if the patient is already iron starved (has low ferritin level). This is consistent with the conclusions of the manuscript that DFO toxicity is mediated by iron starvation. Suggest a) the authors should discuss this clinical recommendation and how it accords with the conclusions of their paper, b) the authors should go back into the clinical histories of their 4 chelated thalassemic patients to see if perhaps they were chelated in a setting in which ferritin was already low.

The data are clear, the paper is well written, and the paper is thoroughly referenced. The conclusions are important and interesting.

Referee #3 (Comments on Novelty/Model System for Author):

Please see "comments to the Authors": certain points of the protocol might be presented and further explained in the main text to facilitate the reading and understanding of the experimental sets; some questions about the clinical part of the study also need clarification.

Referee #3 (Remarks for Author):

The study (manuscript entitled "HIF2α Activation and Mitochondrial Deficit due to Iron Chelation Cause Retinal Atrophy") is based on the documentation of ocular toxicity associated with the clinical use of the iron chelator deferoxamine (DFO). The scientific question is of scientific and clinical interest considering the dual role of iron in the ocular health, namely on the one hand the iron deficiency in the eye can significantly damage the metabolic homeostasis of the retinal pigment epithelial (RPE) and, on the other hand the intracellular accumulation of iron was associated with harmful effects, e.g. oxidative stress, increased levels of pro-inflammatory cholesterol in retina, etc.

Here, the Authors aimed to uncover a target mechanism as a therapeutic solution to DFO-related retinopathy. By suppressing hyper-activity of HIF2α and preserving mitochondrial dysfunction by α-ketoglutarate (AKG) it was found that i) AKG protects the RPE against lesions both in vitro and in vivo; ii) demonstrated a susceptibility of RPE to DFO (compared to neuroretina) and iii) identified upregulation of hypoxia inducible factor (HIF) 2a - a master regulator of oxygen homeostasis and aerobic glycolysis implicated in angiogenesis, extracellular matrix dynamics and cell survival- and mitochondrial deficit as the most prominent pathogenesis underlying the DFO RPE atrophy. Since the AKG supplementation was found to prevent RPE cell death and alleviate patient visual decline due to DFO intake, it was suggested as a possible therapeutic agent against DFO-associated retinopathy.

Comments, questions, suggestions:
"Four patients of ß-thalassemia intermedia with a history of blood transfusion were subject to iron chelation by DFO for at least 16 years": Please specify the retrospective character of the study. The Case IV only had a history of taking AKG (2g/day) as a supplement for 18 months? How this case could be compared to the other three cases (patients who did not receive AKG)? How the protective effect of AKG in this clinical case could specifically attributed to AKG? From only one clinical observation/documentation, could we be sure that the secondary photoreceptor malfunction or degeneration was halted exclusively by AKG? The demographic characteristics of the 4 patients are very different, specifically Case II is only 26 yrs old (though with 17 yrs history of chelation therapy), while Case IV is 64 yrs old (16 yrs history of chelation therapy): could you comment your results considering that iron levels in the retina have been reported increasing with age in human eyes?

"We found a progression of the spotting presentation throughout the course of DFO injection in mice (Figure 2A - C)." The time points could be mentioned also in the main text. Otherwise, the reader has to check the "Material and Method" section to find for "...100 mg/kg DFO three times a week from the weaning age onwards" that "mice were injected with DFO for 10 months...". But then (page 9) we see that "In addition to anatomical changes, we characterized functional impairment of both neuroretina and RPE by ERG on mice injected with DFO for six months." or "mice treated with DFO for five and ten months." Please make clear the time-schedule to avoid confusion.

Some wording modifications could also make the text more readily accessible, e.g. "In order to specify a potential impact of RPE pathology in response to DFO toxicity" may be changed here and in other phrases to "... in response to DFO".

Can you comment the significance/clinical relevance of the findings about "toxic effect of DFO at 10 mM" at different time intervals post treatment?

Regarding the impact of the HIFα pathway on RPE pathology sounds and the comparison between HIF1 and HIF2, please comment other possible mechanisms, if any.

Since AKG was previously reported to halt the progression of retinal degeneration in mice (as mentioned by the Authors: Rowe et al, 2021), please underline the input of your finding to the field.

The discussion (page 19) states that RPE abnormalities have previously been characterized and postulated as primary lesions associated with DFO chelation therapy. Maybe the here-reported results should be emphasized in a comparative manner in order to highlight their novelty.

The potential of transferrin for treatment of retinal pathologies (compared to other chelators or antioxidants) could be mentioned. It would be of interest to mention (introduction or discussion) if other iron chelators provoke retinal toxicity. The Authors should better position their work versus the Dunaieff or Courtois/Behar-Cohen works.

Dear Editor and Reviewers,

We are pleased with the positive response to our manuscript titled, "HIF2α Activation and Mitochondrial Deficit due to Iron Chelation Cause Retinal Atrophy**"** for consideration in *EMBO Molecular Medicine*, and excited by the degree of the reviewers' enthusiasm for our work. We are also grateful for all of the thoughtful and constructive comments.

This letter provides point-by-point responses to the Reviewers' comments. The Reviewers' concerns, shown in regular type, are followed by our response that are underlined:

We hope you will find out responses adequately address all the reviewers' comments and that our submission is acceptable for publication in *EMBO Molecular Medicine.*

Thank you for your consideration.

Best,
Stephen Tsang on behalf of all authors

***** Reviewer's comments and Author Responses*****

Referee #1 (Remarks for Author):

Deferoxamine treatment, an iron chelator can have severe adverse effects, including ocular toxicity.
The group here presents interesting data from patients, a mouse model and iPS-derived RPE cells. The paper reports a specific susceptibility of RPE cells to deferoxamine that leads to mitochondrial (HIF2a) dysfunction leading then to RPE atrophy.

I am very enthusiastic about the first parts of the paper that show a clear translational approach in patients and mice. The last part with a-ketoglutarate (AKG) as a direct therapy for RPE damage via HIF2A inhibition is less clear. There have been several papers that citric cycle intermediates can alleviate retinal degeneration in more general setting of IRDs. This point should be at least discussed carefully.

A: We understand the reviewer's concern and appreciate the suggestions. We provided further characterization of the inhibitory effect of AKG on HIF1α and 2α, including immunoblotting analyses on the RPE from the mice that were subject to chronic treatment of DFO with and without AKG treatment. The data are presented in Figure EV 4 and Figure 7.
Additionally, we cited and discussed the rescuing effect of intermediary metabolites identified previously, which focused on initial screening schemes to identify protective agents and highlighted phenotypic modification (Paragraph 2, Page 17). Phenotypic protection was reported in the neural system (1-3), especially in the context of inherited disorders. This study further validated the rescue effects of AKG in an acquired degenerative disease induced by drug

toxicity, which could be more prevalent in the clinic. Additionally, we highlighted the molecular basis that underpins this protective effect and raised the concern about potential limitations in applying intermediary metabolites for protecting against neurodegeneration.

Minor points:

1. There is a hypothesis that hyperreflective spots seen in mouse retinal fundus imaging of damage models could potentially represent reactive immune cells (microglia). It would be worth to have a look on retinal and RPE/choroidal flat mounts stained with markers such as Iba1 to rule out this immune-mediated phenomenon in the mouse damage/treatment studies.
   A: We tested for the presence of microglia in RPE flat mount harvested from the mice that received DFO treatment for 10 months. The age-matched untreated mice were used as the control. Based on our results, IBA1-positive cells were scarcely present in the DFO-treated mice, which is comparable to the untreated controls. The data have been included in Appendix Figure S1A - C.
2. Fig. 2K-N. The phalloidin staining of the (damaged) RPE cell network should be supplemented with a second verification marker such as zonula occludens protein 1 (ZO1) (as has been done for in vitro RPE in Fig. 3).
   A: As the reviewer advised, we included the IHC for ZO1 in RPE flat mounts collected from the mice with more than 10 months of DFO treatment. DFO-treated RPE appeared polymorphous and lost the *hexagonal* packing compared with the untreated controls. Please see Appendix Figure S1D and E.
3. I wonder whether the strongly induced expression in the iRPE cells in VEGF transcripts really translates to VEGF-A protein expression, which would also indicate a proangiogenic trigger of deferoxamine.
   A: Thanks for the intriguing question. We measured the secretion of VEGF-A in the DFO-treated iRPE cells. As anticipated, an elevation of secreted VEGF-A was present but not significant, 24 hours post-DFO treatment. Whereas a significant increase in VEGF-A was detected in the iRPE cells with DFO treatment for 48 hours. In fact, increased VEGF-A due to DFO stimulation was previously reported (4). Please find the analyses added in Appendix Figure S1F and G.

Referee #2 (Comments on Novelty/Model System for Author):

The major conclusions need to be confirmed in the mouse model. The iRPE data are of unclear validity due to the extremely high concentration of DFO used.

A: We are grateful for the reviewer's recommendation and we validated phenotypic changes associated with DFO toxic effect in the mice (Figures 2 and 6). As a major finding, we tested HIFα levels in mouse RPE, which corroborated our *in-vitro* observations. The data are presented in Figure 7H and Figure EV 4F.

Admittedly, recapitulating phenotypic changes with mouse RPE, especially at the molecular level is challenging. Several aspects of modeling and technical difficulties hinder us from making direct interpretations, including:

1. Unlike genetically engineered models that are easier to yield consistent outcomes, tissues collected from pharmacological-induced mouse models via systemic delivery are volatile and lack biochemical stability for post-mortem examinations, which is exemplified by the abundant degradation of HIFα tested in our mouse RPE.
2. Isolation of RPE from the back of the eye allows for contamination by other types of retinal cells, as well as the choroid. These interfere with the outcomes, especially in consideration of making claims about different metabolic systems in the neuroretina.
3. We attempted to delineate the metabolic profiles of the RPE under the influence of DFO as we identified *in vitro*. Monolayered RPE, harvested from the mice treated by DFO with or without AKG, was used for the Extracellular Flux Seahorse assay by following the protocol for testing mitochondrial functions with frozen tissues. However, our results failed to reveal statistical significance among the groups of untreated, DFO treated and DFO treated with AKG supplementation due to variation. It should be noted that seahorse is a less sensitive approach to capturing delicate changes in the animal tissue. Handling of tissue dissection and availability of frozen tissues could be even less sufficient to detect transient and subtle alterations in mitochondria as well as their interplay with aerobic glycolysis.

This test has been completed. We are willing to share it upon the reviewers' request.

Referee #2 (Remarks for Author):

In this article, the authors address the problem of Deferoxamine (DFO) toxicity on the eye. DFO is an iron chelator in clinical use to remove iron from overloaded patients with thalassemia, especially those dependent on red cell transfusions. Ocular toxicity occurs in less than 10% of patients taking DFO, and it is unclear why they are particularly susceptible and what the mechanism of toxicity is. The manuscript presents clinical data from 4 patients, mouse model experiments and iRPE experiments. The authors come to the surprising conclusion that the toxic effects of DFO are due to iron starvation of the RPE, manifested in particular by mitochondrial respiratory dysfunction and HIF2 alpha induction. The toxic effects could be partially rescued by treatment with alpha keto glutarate, a PHD substrate involved in HIF induction.

Pharmacology - How were the DFO doses used arrived at? The dose for the mice was 100 mg/kg, somewhat higher than the recommended dose for human use, but in the same range. What is the effective drug concentration here? The dose for the iRPE experiments, 100 mM, seems to be a huge excess over the recommended dosing. Toxicity from this high dose may or may not reflect the situation in intact retina in an organism (mouse or human) treated with the recommended DFO dosing. The conclusions regarding the role of mitochondrial respiration and HIF2 alpha induction in DFO toxicity are based on the experiments with iRPE with the extra high DFO concentration. If possible these results should be confirmed in the mouse model. Does the RPE tested in seahorse lose respiratory capacity? Is HIF2 alpha induced in RPE of the mouse treated with DFO. Does AKG rescue in the mouse setting.

A: Indeed, the concentration used in this study, especially the one for *in-vitro* assays is much higher than is frequently used in other research studies. We chose this high dosage as we desired to accelerate phenotypic manifestations in iRPE, since DFO toxicity takes more than 10 years to

develop in human patients. We included this information in the manuscript (Paragraph 2, Page 9). For choosing the dosage in the mice, we referred to the clinical dosage for inducing toxicity in order to simulate the progression of the phenotypes in the most efficient manner. Meanwhile subtle or transient changes can be captured within a reasonable duration of time.

The *in-vivo* phenotyping of DFO and AKG-treated mice was included in Figure 2 and Figure 6. At the molecular level, we included immunoblotting against HIFα in mouse RPE (Figure 7H) as further evidence of hyperactivation of HIF2α by DFO, which can be suppressed by AKG. As aforementioned, we attempted to recapitulate the suppressive effects of DFO on mitochondrial respiration using the seahorse assay. The results lack significant differences. This is likely due to the fact that some mice remained robustly resistant to the toxic effect even at a high dosage, similar to the variation observed in human patients taking DFO We touched upon part to the manuscript (Paragraph 2, Page 19).

A large proportion of molecular changes *in vitro* take place at a 24-hour time point while gross phenotypic changes were hardly seen at this time. The 48-hour time point appears to be more compelling in exhibiting phenotypes. Additionally, we were aware that DFO is conventionally regarded as being pro-survival in cells undergoing ferroptosis. However, high-dose DFO, despite being less investigated, has been reported to induce the death of cancer cells, which corroborates our findings. Additionally, the guideline, based on *UpToDate*, documented such toxicity at a high dose as well based on the reviewer's feedback, which further supports our findings in this study. We have revised the text and addressed the differential impact of DFO at different doses in the discussion section (Paragraph 1, Page 16).

A major finding of the paper is that the toxicity of DFO relates to iron starvation. In the write-up on Deferoxamine (Drug information, Up-to-Date), it says "Ocular disturbances ...have been reported following prolonged administration at high doses or in patients with low ferritin levels..." The database goes on to say, "a lower dose may be required if ferritin levels are low. In general, the therapeutic index should be kept at <0.05. Therapeutic index = mean daily deferoxamine dose (mg/kg)/ferritin (mcg/L)." The implication of this recommendation is that DFO toxicity may occur if the patient is already iron starved (has low ferritin level). This is consistent with the conclusions of the manuscript that DFO toxicity is mediated by iron starvation. Suggest a) the authors should discuss this clinical recommendation and how it accords with the conclusions of their paper, b) the authors should go back into the clinical histories of their 4 chelated thalassemic patients to see if perhaps they were chelated in a setting in which ferritin was already low.

A: We appreciated the reviewer's comment about clinical guidance for the precautions of the usage of DFO. We revisited our patients' blood test results. By referring to relevant publications and consulting hematologists, we found that serum ferritin higher than the normal range is common in transfusion-dependent patients. Typically, physicians are less likely to to reduce the elevated level of serum ferritin to the normal range for these patients in the clinic. Intriguingly, we learned that ferritin assays lack specificity and sensitivity, being subject to systemic conditions such as inflammatory reactions, etc. Reliable examinations, including MRI and invasive tissue biopsy, need to be considered if local iron overload is highly suspected. We have

addressed this in the discussion section (Paragraph 2, Page 19) and included patients' ferritin levels over the years in Appendix Table S1.

The data are clear, the paper is well written, and the paper is thoroughly referenced. The conclusions are important and interesting.

Referee #3 (Comments on Novelty/Model System for Author):

Please see "comments to the Authors": certain points of the protocol might be presented and further explained in the main text to facilitate the reading and understanding of the experimental sets; some questions about the clinical part of the study also need clarification.

Referee #3 (Remarks for Author):

The study (manuscript entitled "HIF2α Activation and Mitochondrial Deficit due to Iron Chelation Cause Retinal Atrophy") is based on the documentation of ocular toxicity associated with the clinical use of the iron chelator deferoxamine (DFO). The scientific question is of scientific and clinical interest considering the dual role of iron in the ocular health, namely on the one hand the iron deficiency in the eye can significantly damage the metabolic homeostasis of the retinal pigment epithelial (RPE) and, on the other hand the intracellular accumulation of iron was associated with harmful effects, e.g. oxidative stress, increased levels of pro-inflammatory cholesterol in retina, etc.

Here, the Authors aimed to uncover a target mechanism as a therapeutic solution to DFO-related retinopathy. By suppressing hyper-activity of HIF2α and preserving mitochondrial dysfunction by α-ketoglutarate (AKG) it was found that i) AKG protects the RPE against lesions both in vitro and in vivo; ii) demonstrated a susceptibility of RPE to DFO (compared to neuroretina) and iii) identified upregulation of hypoxia inducible factor (HIF) 2a - a master regulator of oxygen homeostasis and aerobic glycolysis implicated in angiogenesis, extracellular matrix dynamics and cell survival- and mitochondrial deficit as the most prominent pathogenesis underlying the DFO RPE atrophy. Since the AKG supplementation was found to prevent RPE cell death and alleviate patient visual decline due to DFO intake, it was suggested as a possible therapeutic agent against DFO-associated retinopathy.

Comments, questions, suggestions:
"Four patients of ß-thalassemia intermedia with a history of blood transfusion were subject to iron chelation by DFO for at least 16 years": Please specify the retrospective character of the study. The Case IV only had a history of taking AKG (2g/day) as a supplement for 18 months? How this case could be compared to the other three cases (patients who did not receive AKG)? How the protective effect of AKG in this clinical case could specifically attributed to AKG? From only one clinical observation/documentation, could we be sure that the secondary photoreceptor malfunction or degeneration was halted exclusively by AKG? The demographic characteristics of the 4 patients are very different, specifically Case II is only 26 yrs old (though with 17 yrs history of chelation therapy), while Case IV is 64 yrs old (16 yrs history of chelation

therapy): could you comment your results considering that iron levels in the retina have been reported increasing with age in human eyes?

A: We appreciate the reviewer's careful reading of our manuscript. Based on the reviewer's suggestion, we emphasized a retrospective review of the patient's ophthalmic presentations in the result section (Paragraph 1, Page 7). Furthermore, we would like to clarify that potential amelioration in Case IV was compared to himself before intake of AKG *only*, which is an observation in a longitudinal manner, and we made sure not to overstate the claim on whether the protection is a direct result of AKG supplementation. Admittedly, the cohort overall is small. Patients at the stage of ophthalmic presentations in the clinic are rare. Therefore, it is difficult to identify a large number of patients with orphan disorders to conduct a systematic clinical review, which sufficiently highlights the significance of our work by using *in vitro* and *in vivo* models. We have noted this limitation in the discussion section (Paragraph 2, Page 19). Excitingly, we observed protection against morphological damage due to DFO toxicity as well as mitigation of neuroretinal degeneration by AKG.

In terms of the fluctuation of iron in the retina, we also read the report about the accumulation of iron with age in the human population. However, the ophthalmic presentations in the patients in this study lack common and classic signs of iron accumulation. We have elucidated this regard in both the result and discussion sections as the reviewer recommended (Paragraph 1, Page 5; Paragraph 1, Page 16).

"We found a progression of the spotting presentation throughout the course of DFO injection in mice (Figure 2A - C)." The time points could be mentioned also in the main text. Otherwise, the reader has to check the "Material and Method" section to find for "...100 mg/kg DFO three times a week from the weaning age onwards" that "mice were injected with DFO for 10 months...". But then (page 9) we see that "In addition to anatomical changes, we characterized functional impairment of both neuroretina and RPE by ERG on mice injected with DFO for six months." or "mice treated with DFO for five and ten months." Please make clear the time-schedule to avoid confusion.

A: We thank the reviewer for the meticulous comments and have clarified the timeline throughout the manuscript. The regimen for DFO administration is mentioned in the results section. We used various time points to sufficiently induce desired phenotypes as well as most availability of experimental animals. These have been included both in the figures and in the main text based on the reviewer's suggestions (Paragraph 2, Page 7; Paragraphs 1 & 2, Page 8; Paragraph 2, Page 13; Paragraph 1, Page 14; Figure 6E – M; N and O).

Some wording modifications could also make the text more readily accessible, e.g. "In order to specify a potential impact of RPE pathology in response to DFO toxicity" may be changed here and in other phrases to "... in response to DFO".'

A: We appreciate the reviewer's suggestion and have modified the wording accordingly to minimize partial interpretations of the results.

Can you comment the significance/clinical relevance of the findings about "toxic effect of DFO at 10 (100?) mM" at different time intervals post treatment?

A: We thank the reviewer for raising this point. In fact, we detected the *in vitro* and *in vivo* manifestations linked to DFO's toxic effects at different time points. Fundamental molecular changes tend to be initiated at an earlier time point while gross phenotypical changes are not manifested until a later time point, which implies a drastic disruption has taken place long before clinical manifestations. This reflects the patients' conditions, where DFO toxicity is a prolonged and accumulative process. Additionally, we found that the rescuing effects could be optimized if AKG was applied at an earlier time point. AKG is insufficient to reverse the course of DFO-related damage as the toxic effects accumulate over time. Clinically, we believe that DFO toxicity should be monitored regularly at an early stage even before the appearance of pathological hyper-fluorescent lesions. Therapeutic interventions should be employed at an early stage or even as a preventive measure. We have noted it in the discussion section (Paragraph 2, Page 17; Paragraph 2, Page 19).

Regarding the impact of the HIFα pathway on RPE pathology sounds and the comparison between HIF1 and HIF2, please comment other possible mechanisms, if any.

A: The core concept of this study is the perturbation of iron homeostasis due to DFO chelation. Any iron-dependent biochemical reactions or enzymatic activities could be impacted, such as dysfunctional cytochrome c, production of oxidative stress and its sources, etc. Notably, HIFα serves as a master regulator in the majority of these changes.

We have added information about these potential mechanistic explanations for the RPE damage associated with DFO toxicity in the discussion section (Paragraph 2, Page 17).

Since AKG was previously reported to halt the progression of retinal degeneration in mice (as mentioned by the Authors: Rowe et al, 2021), please underline the input of your finding to the field.

A: We thank the reviewer for the suggestion, and have further highlighted the significance of our study in relation to the previous investigations (Paragraph 1, Page 16; Paragraph 2, Page 17).

The discussion (page 19) states that RPE abnormalities have previously been characterized and postulated as primary lesions associated with DFO chelation therapy. Maybe the here-reported results should be emphasized in a comparative manner to highlight their novelty.

A: As the reviewer recommended, the significance of this study was highlighted as a mechanistic investigation of DFO-related retinopathy with clinic relevance. More importantly, our finding pinpointed HIF2α as a key player and characterized the metabolic profile involved in the RPE pathology that could be extrapolated to a greater scope of neurodegenerative disorders, such as age-related macular degeneration.

The potential of transferrin for the treatment of retinal pathologies (compared to other chelators or antioxidants) could be mentioned. It would be of interest to mention (introduction or

discussion) if other iron chelators provoke retinal toxicity. The Authors should better position their work versus the Dunaieff or Courtois/Behar-Cohen works.

A: Based on the reviewer's comments, we carefully addressed similarities and differences between DFO from its counterparts, such as DFP in biological properties and pharmacological toxicity. (Paragraph 1, Pages 16; Paragraph 2, Page 17). Additionally, we plan to conduct further experiments to explore this question in future studies.

References:

1. A. Y. Kim, E. J. Baik, Glutamate Dehydrogenase as a Neuroprotective Target Against Neurodegeneration. *Neurochem Res* **44**, 147-153 (2019).
2. F. K. Odorcyk *et al.*, Differential glucose and beta-hydroxybutyrate metabolism confers an intrinsic neuroprotection to the immature brain in a rat model of neonatal hypoxia ischemia. *Exp Neurol* **330**, 113317 (2020).
3. K. Sawa *et al.*, Krebs Cycle Intermediates Protective against Oxidative Stress by Modulating the Level of Reactive Oxygen Species in Neuronal HT22 Cells. *Antioxidants (Basel)* **6** (2017).
4. E. A. Wahl, T. L. Schenck, H. G. Machens, E. R. Balmayor, VEGF released by deferoxamine preconditioned mesenchymal stem cells seeded on collagen-GAG substrates enhances neovascularization. *Sci Rep* **6**, 36879 (2016).

30th Nov 2022

Dear Dr. Tsang,

Thank you for the submission of your manuscript to EMBO Molecular Medicine. We have now received feedback from the three referees who originally reviewed your manuscript. As you will see below, they are now supportive of publication, and I am therefore pleased to inform you that we will be able to accept your manuscript once the following editorial points will be addressed:

1/ Manuscript text:
- Please address the queries from our data editor in the Data edited manuscript file (figure legends). Please accept the previous changes and only keep in track changes mode any new modification, including the responses to the data editor.
- Please provide up to 5 keywords.
- Material and methods:
o iRPE cells: include a statement that the experiments conformed to the principles set out in the WMA Declaration of Helsinki and the Department of Health and Human Services Belmont Report.
o Provide the antibody dilutions.
- Acknowledgements: Please make sure that the funding information provided in the manuscript is the same as what is provided in the submission system.
- Author contributions: CRediT has replaced the traditional author contributions section because it offers a systematic machine-readable author contributions format that allows for more effective research assessment. Please remove the Authors Contributions from the manuscript and use the free text boxes beneath each contributing author's name in our system to add specific details on the author's contribution. More information is available in our guide to authors.
- Please remove the Appendix legends from the main manuscript file.

2/ Figures and Appendix:
- Tables EV1 and EV2 should be renamed Table 1 and 2, or uploaded as separate files.
- The appendix figure and table should be merged in one PDF file with a table of content. Legends should be added. The nomenclature should be corrected to Appendix Figure S1 and Appendix Table S1.

3/ Thank you for providing Source Data. In Source Data for Fig. 7H, the blot for HIF2a seems upside down compared to the figure presented in the manuscript, please check. The same applies to Source Data Fig. EV4, Actin blot.

4/ Please provide "The paper explained": EMBO Molecular Medicine articles are accompanied by a summary of the articles to emphasize the major findings in the paper and their medical implications for the non-specialist reader. Please provide a draft summary of your article highlighting
- the medical issue you are addressing,
- the results obtained and
- their clinical impact.
This may be edited to ensure that readers understand the significance and context of the research. Please refer to any of our published articles for an example.

5/ Synopsis:
Thank you for providing a nice synopsis image. I resized it to fit our format, please let me know if you approve the final image (attached).
I also slightly edited the text, please let me know if you agree with the following or amend as you see fit:
Deferoxamine (DFO) chelation therapy stabilizes HIF2α and disrupts mitochondrial oxidative phosphorylation, leading to RPE atrophy. Ablating HIF2α and preserving mitochondrial oxidative phosphorylation by α-ketoglutarate (AKG) mitigates RPE cell death, which ameliorates visual impairment in the clinic.
- DFO primarily acts on RPE to disrupt iron homeostasis causing atrophic lesions.
- DFO upregulates HIF2α and undermines mitochondrial oxidative phosphorylation in the RPE, accounting for its susceptibility to iron removal.
- RPE intolerance to HIF2α-induced anaerobic glycolysis contributes to susceptibility to DFO toxicity. RPE survival is sustained by mitochondrial oxidative phosphorylation.
- AKG, an intermediary metabolite of the Krebs cycle, destabilizes HIF2α and preserves mitochondrial respiration capacity, which protects RPE from damage and mitigates visual impairment in the clinic.
6/ As part of the EMBO Publications transparent editorial process initiative (see our Editorial at http://embomolmed.embopress.org/content/2/9/329), EMBO Molecular Medicine will publish online a Review Process File (RPF) to accompany accepted manuscripts.
This file will be published in conjunction with your paper and will include the anonymous referee reports, your point-by-point response and all pertinent correspondence relating to the manuscript. Let us know whether you agree with the publication of the RPF and as here, if you want to remove or not any figures from it prior to publication.

Please note that the Authors checklist will be published at the end of the RPF.

I look forward to receiving your revised manuscript.

Yours sincerely,

Lise Roth

Lise Roth, PhD
Senior Editor
EMBO Molecular Medicine

***** Reviewer's comments *****

Referee #1 (Remarks for Author):

The authors have carefully addressed my previous concerns. I have not further questions.

Referee #2 (Comments on Novelty/Model System for Author):
Authors have done extensive new work and reediting and the manuscript is much improved. To be honest, I am still troubled by the use of such high Desferal concentrations in the iRPE cell model system, which constitutes a large portion of the presented data. Authors have addressed this problem by including more mouse experiments and a dose titration of an iRPE experiment with toxicity readout. Thank you for digging out the ferritin data on the chelated patients, I think it is useful to include.

Referee #3 (Remarks for Author):

The revised version of the manuscript goes further in deciphering the mechanisms of ocular toxicity associated with the use of deferoxamine (DFO), an iron chelator in clinical use for decades. The results obtained point, with more precision now, on the molecular basis playing role in the susceptibility of RPE cells to DFO and suggest pathways for rescuing mitochondrial function and visual function in patients.

The quality of the revised manuscript appears significantly improved, the additional references are relevant, the clinical/translational potential of the findings is well-defined.

The authors addressed the minor editorial issues.

13th Dec 2022

Dear Dr. Tsang,

Thank you for providing the revised files. I am pleased to inform you that your manuscript is now accepted for publication in EMBO Molecular Medicine and will be sent to our publisher to be included in the next available issue.

Please note that the bullet points from the synopsis must not exceed 30 words each, I therefore had to slightly modify your synopsis. Please inform us immediately if you do not agree with the following:

Deferoxamine (DFO) stabilizes HIF2α and disrupts mitochondrial oxidative phosphorylation, leading to RPE atrophy. Inhibiting HIF2α and preserving mitochondrial oxidative phosphorylation by α-ketoglutarate (AKG) mitigates RPE cell death, which ameliorates visual impairment in the clinic.
- DFO primarily acts on RPE and disrupts its iron homeostasis, causing atrophic lesions.
- DFO upregulates HIF2α and undermines mitochondrial oxidative phosphorylation in the RPE, accounting for its susceptibility to iron depletion.
- RPE intolerance to HIF2α-induced anaerobic glycolysis contributes to susceptibility to DFO toxicity.
- RPE survival is sustained by mitochondrial OXPHOS, whereas the anaerobic glycolytic photoreceptor is initially spared from enhanced HIF levels.
- AKG, an intermediary metabolite of the Krebs cycle, destabilizes HIF2α and preserves mitochondrial respiration capacity, which protects RPE from damage and mitigates visual impairment in the clinic.

Please read below for additional IMPORTANT information regarding your article, its publication and the production process.

Congratulations on your interesting work!

With kind regards,

Lise Roth

Lise Roth, Ph.D
Senior Editor
EMBO Molecular Medicine

Follow us on Twitter @EmboMolMed
Sign up for eTOCs at embopress.org/alertsfeeds

*** *** *** IMPORTANT INFORMATION *** *** ***

SPEED OF PUBLICATION
The journal aims for rapid publication of papers, using using the advance online publication "Early View" to expedite the process: A properly copy-edited and formatted version will be published as "Early View" after the proofs have been corrected. Please help the Editors and publisher avoid delays by providing e-mail address(es), telephone and fax numbers at which author(s) can be contacted.

Should you be planning a Press Release on your article, please get in contact with embomolmed@wiley.com as early as possible, in order to coordinate publication and release dates.

LICENSE AND PAYMENT:

All articles published in EMBO Molecular Medicine are fully open access: immediately and freely available to read, download and share.

EMBO Molecular Medicine charges an article processing charge (APC) to cover the publication costs. You, as the corresponding author for this manuscript, should have already received a quote with the article processing fee separately. Please let us know in case this quote has not been received.

Once your article is at Wiley for editorial production you will receive an email from Wiley's Author Services system, which will ask you to log in and will present you with the publication license form for completion. Within the same system the publication fee can be paid by credit card, an invoice, pro forma invoice or purchase order can be requested.

Payment of the publication charge and the signed Open Access Agreement form must be received before the article can be published online.

PROOFS

You will receive the proofs by e-mail approximately 2 weeks after all relevant files have been sent o our Production Office. Please return them within 48 hours and if there should be any problems, please contact the production office at embopressproduction@wiley.com.

Please inform us if there is likely to be any difficulty in reaching you at the above address at that time. Failure to meet our deadlines may result in a delay of publication.

All further communications concerning your paper proofs should quote reference number EMM-2022-16525-V3 and be directed to the production office at embopressproduction@wiley.com.

EMBO Press Author Checklist

Corresponding Author Name: Stephen H. Tsang	
Journal Submitted to: EMBO Molecular Medicine	
Manuscript Number: EMM-2022-16525	

USEFUL LINKS FOR COMPLETING THIS FORM
The EMBO Journal - Author Guidelines
EMBO Reports - Author Guidelines
Molecular Systems Biology - Author Guidelines
EMBO Molecular Medicine - Author Guidelines

Reporting Checklist for Life Science Articles (updated January 2022)
This checklist is adapted from Materials Design Analysis Reporting (MDAR) Checklist for Authors. MDAR establishes a minimum set of requirements in transparent reporting in the life sciences (see Statement of Task: 10.31222/osf.io/9sm4x). Please follow the journal's guidelines in preparing your manuscript.
Please note that a copy of this checklist will be published alongside your article.

Abridged guidelines for figures
1. Data
The data shown in figures should satisfy the following conditions:
- the data were obtained and processed according to the field's best practice and are presented to reflect the results of the experiments in an accurate and unbiased manner.
- ideally, figure panels should include only measurements that are directly comparable to each other and obtained with the same assay.
- plots include clearly labeled error bars for independent experiments and sample sizes. Unless justified, error bars should not be shown for technical replicates.
- if n<5, the individual data points from each experiment should be plotted. Any statistical test employed should be justified.
- Source Data should be included to report the data underlying figures according to the guidelines set out in the authorship guidelines on Data Presentation.

2. Captions
Each figure caption should contain the following information, for each panel where they are relevant:
- a specification of the experimental system investigated (eg cell line, species name).
- the assay(s) and method(s) used to carry out the reported observations and measurements.
- an explicit mention of the biological and chemical entity(ies) that are being measured.
- an explicit mention of the biological and chemical entity(ies) that are altered/varied/perturbed in a controlled manner.
- the exact sample size (n) for each experimental group/condition, given as a number, not a range;
- a description of the sample collection allowing the reader to understand whether the samples represent technical or biological replicates (including how many animals, litters, cultures, etc.).
- a statement of how many times the experiment shown was independently replicated in the laboratory.
- definitions of statistical methods and measures:
  - common tests, such as t-test (please specify whether paired vs. unpaired), simple χ2 tests, Wilcoxon and Mann-Whitney tests, can be unambiguously identified by name only, but more complex techniques should be described in the methods section;
  - are tests one-sided or two-sided?
  - are there adjustments for multiple comparisons?
  - exact statistical test results, e.g., P values = x but not P values < x;
  - definition of 'center values' as median or average;
  - definition of error bars as s.d. or s.e.m.

Please complete ALL of the questions below.
Select "Not Applicable" only when the requested information is not relevant for your study.

Materials

Newly Created Materials	Information included in the manuscript?	In which section is the information available? (Reagents and Tools Table, Materials and Methods, Figures, Data Availability Section)
New materials and reagents need to be available; do any restrictions apply?	Not Applicable	

Antibodies	Information included in the manuscript?	In which section is the information available? (Reagents and Tools Table, Materials and Methods, Figures, Data Availability Section)
For **antibodies** provide the following information:<br>- Commercial antibodies: RRID (if possible) or supplier name, catalogue number and or/clone number<br>- Non-commercial: RRID or citation	Yes	Materials and Methods

DNA and RNA sequences	Information included in the manuscript?	In which section is the information available? (Reagents and Tools Table, Materials and Methods, Figures, Data Availability Section)
**Short novel DNA or RNA including primers, probes**: provide the sequences.	Yes	Table EV2

Cell materials	Information included in the manuscript?	In which section is the information available? (Reagents and Tools Table, Materials and Methods, Figures, Data Availability Section)
**Cell lines:** Provide species information, strain. Provide accession number in repository **OR** supplier name, catalog number, clone number, and/**OR** RRID.	Not Applicable	
**Primary cultures:** Provide species, strain, sex of origin, genetic modification status.	Not Applicable	
Report if the cell lines were recently **authenticated** (e.g., by STR profiling) and tested for mycoplasma contamination.	Yes	Materials and Methods

Experimental animals	Information included in the manuscript?	In which section is the information available? (Reagents and Tools Table, Materials and Methods, Figures, Data Availability Section)
**Laboratory animals or Model organisms:** Provide species, strain, sex, age, genetic modification status. Provide accession number in repository **OR** supplier name, catalog number, clone number, **OR** RRID.	Yes	Materials and Methods
**Animal observed in or captured from the field:** Provide species, sex, and age where possible.	Not Applicable	
Please detail **housing and husbandry conditions**.	Yes	Materials and Methods

Plants and microbes	Information included in the manuscript?	In which section is the information available? (Reagents and Tools Table, Materials and Methods, Figures, Data Availability Section)
**Plants:** provide species and strain, ecotype and cultivar where relevant, unique accession number if available, and source (including location for collected wild specimens).	Not Applicable	
**Microbes:** provide species and strain, unique accession number if available, and source.	Not Applicable	

Human research participants	Information included in the manuscript?	In which section is the information available? (Reagents and Tools Table, Materials and Methods, Figures, Data Availability Section)
If collected and within the bounds of privacy constraints report on age, sex and gender or ethnicity for all study participants.	Yes	Table EV1

Core facilities	Information included in the manuscript?	In which section is the information available? (Reagents and Tools Table, Materials and Methods, Figures, Data Availability Section)
If your work benefited from core facilities, was their service mentioned in the acknowledgments section?	Yes	Acknowledgement

Design

Study protocol	Information included in the manuscript?	In which section is the information available? (Reagents and Tools Table, Materials and Methods, Figures, Data Availability Section)
If study protocol has been **pre-registered, provide DOI in the manuscript**. For clinical trials, provide the trial registration number **OR** cite DOI.	Not Applicable	
Report the **clinical trial registration number** (at ClinicalTrials.gov or equivalent), where applicable.	Not Applicable	

Laboratory protocol	Information included in the manuscript?	In which section is the information available? (Reagents and Tools Table, Materials and Methods, Figures, Data Availability Section)
Provide DOI OR other citation details if **external detailed step-by-step protocols** are available.	Yes	Materials and Methods

Experimental study design and statistics	Information included in the manuscript?	In which section is the information available? (Reagents and Tools Table, Materials and Methods, Figures, Data Availability Section)
Include a statement about **sample size** estimate even if no statistical methods were used.	Yes	Figure legends
Were any steps taken to minimize the effects of subjective bias when allocating animals/samples to treatment (e.g. **randomization procedure**)? If yes, have they been described?	Not Applicable	
Include a statement about **blinding** even if no blinding was done.	Not Applicable	
Describe **inclusion/exclusion criteria** if samples or animals were excluded from the analysis. Were the criteria pre-established?<br><br>If sample or data points were omitted from analysis, report if this was due to attrition or intentional exclusion and provide justification.	Yes	Methods and Materials
For every figure, are **statistical tests** justified as appropriate? Do the data meet the assumptions of the tests (e.g., normal distribution)? Describe any methods used to assess it. Is there an estimate of variation within each group of data? Is the variance similar between the groups that are being statistically compared?	Yes	Figure legends, Methods and Materials

Sample definition and in-laboratory replication	Information included in the manuscript?	In which section is the information available? (Reagents and Tools Table, Materials and Methods, Figures, Data Availability Section)
In the figure legends: state number of times the experiment was **replicated** in laboratory.	Yes	Figure Legends
In the figure legends: define whether data describe **technical or biological replicates**.	Yes	Figure Legends, Methods and Materials

Ethics

Ethics	Information included in the manuscript?	In which section is the information available? (Reagents and Tools Table, Materials and Methods, Figures, Data Availability Section)
Studies involving **human participants**: State details of **authority granting ethics approval** (IRB or equivalent committee(s), provide reference number for approval.	Yes	Materials and Methods
Studies involving **human participants**: Include a statement confirming that **informed consent** was obtained from all subjects and that the experiments conformed to the principles set out in the WMA Declaration of Helsinki and the Department of Health and Human Services Belmont Report.	Yes	Materials and Methods
Studies involving **human participants:** For publication of **patient photos**, include a statement confirming that consent to publish was obtained.	Not Applicable	
Studies involving experimental **animals**: State details of **authority granting ethics approval** (IRB or equivalent committee(s), provide reference number for approval. Include a statement of compliance with ethical regulations.	Yes	Materials and Methods
Studies involving **specimen and field samples:** State if relevant **permits** obtained, provide details of authority approving study; if none were required, explain why.	Not Applicable	

Dual Use Research of Concern (DURC)	Information included in the manuscript?	In which section is the information available? (Reagents and Tools Table, Materials and Methods, Figures, Data Availability Section)
Could your study fall under dual use research restrictions? Please check biosecurity documents and list of **select agents and toxins** (CDC): https://www.selectagents.gov/sat/list.htm	Not Applicable	
If you used a select agent, is the security level of the lab appropriate and reported in the manuscript?	Not Applicable	
If a study is subject to dual use research of concern regulations, is the name of the **authority granting approval and reference number** for the regulatory approval provided in the manuscript?	Not Applicable	

Reporting

The MDAR framework recommends adoption of discipline-specific guidelines, established and endorsed through community initiatives. Journals have their own policy about requiring specific guidelines and recommendations to complement MDAR.

Adherence to community standards	Information included in the manuscript?	In which section is the information available? (Reagents and Tools Table, Materials and Methods, Figures, Data Availability Section)
State if relevant guidelines or checklists (e.g., **ICMJE, MIBBI, ARRIVE, PRISMA**) have been followed or provided.	Not Applicable	
For **tumor marker prognostic studies,** we recommend that you follow the **REMARK** reporting guidelines (see link list at top right). See author guidelines, under 'Reporting Guidelines'. Please confirm you have followed these guidelines.	Not Applicable	
For **phase II and III randomized controlled trials**, please refer to the **CONSORT** flow diagram (see link list at top right) and submit the CONSORT checklist (see link list at top right) with your submission. See author guidelines, under 'Reporting Guidelines'. Please confirm you have submitted this list.	Not Applicable	

Data Availability

Data availability	Information included in the manuscript?	In which section is the information available? (Reagents and Tools Table, Materials and Methods, Figures, Data Availability Section)
Have **primary datasets** been deposited according to the journal's guidelines (see 'Data Deposition' section) and the respective accession numbers provided in the Data Availability Section?	Not Applicable	
Were **human clinical and genomic datasets** deposited in a public access-controlled repository in accordance to ethical obligations to the patients and to the applicable consent agreement?	Not Applicable	
Are **computational models** that are central and integral to a study available without restrictions in a machine-readable form? Were the relevant accession numbers or links provided?	Not Applicable	
If publicly available data were reused, provide the respective **data citations in the reference list**.	Yes	References
